# Cross talk between acetylation and methylation regulators reveals histone modifier expression patterns posing prognostic and therapeutic implications on patients with colon cancer

**DOI:** 10.1186/s13148-022-01290-y

**Published:** 2022-05-23

**Authors:** Rui Zhou, Fuli Xie, Kuncai Liu, Xuee Zhou, Xuemei Chen, Jinzhang Chen, Shaoyan Xi, Zhenhua Huang, Xiaoxiang Rong

**Affiliations:** 1 Department of Oncology, Nanfang Hospital, Southern Medical University, Guangzhou, 510515 Guangdong People’s Republic of China; 2 Department of Radiotherapy, Sun Yat-sen University Cancer Center, Guangzhou, Guangdong People’s Republic of China; 3 State Key Laboratory of Organ Failure Research, Guangdong Provincial Key Laboratory of Viral Hepatitis Research, Department of Hepatology Unit and Infectious Diseases, Nanfang Hospital, Southern Medical University, Guangzhou, Guangdong People’s Republic of China; 4 Department of Pathology, Sun Yat-sen University Cancer Center, State Key Laboratory of Oncology in South China, Collaborative Innovation Center for Cancer Medicine, Guangzhou, 510515 Guangdong People’s Republic of China

**Keywords:** Histone modifications, Colon cancer, Prognosis, Fluorouracil, Chemotherapy, CRISPR library screen, ZEB2

## Abstract

**Background:**

Alterations in histone modifications have been reported to be related to tumorigenicity and tumor progression. However, whether histone modification can aid the classification of patients or influence clinical behavior in patients with colon cancer remains unclear. Therefore, this study aimed to evaluate histone modifier expression patterns using the unsupervised clustering of the transcriptomic expressions of 88 histone acetylation and methylation regulators.

**Results:**

In this study, by consensus clustering analysis based on the transcriptome data of 88 histone modification regulators, we identified four distinct expression patterns of histone modifiers associated with different prognoses, intrinsic fluorouracil sensitivities, biological pathways, and tumor microenvironment characteristics among 1372 colon cancer samples. In these four clusters, the HMC4 cluster represented a stroma activation phenotype characterized by both the worst prognosis and lowest response rates to fluorouracil treatment. Then, we established a scoring scheme comprising 155 genes designated as “HM_score” by using the Boruta algorithm to distinguish colon cancer patients within the HMC4 cluster. Patients with a high HM_score were considered to have high stromal pathway activation, high stromal fraction, and an unfavorable prognosis. Further analyses indicated that a high HM_score also correlated with reduced therapeutic benefits from fluorouracil chemotherapy. Moreover, through CRISPR library screening, ZEB2 was found to be a critical driver gene that mediates fluorouracil resistance, which is associated with histone modifier expression patterns.

**Conclusions:**

This study highlights that characterizing histone modifier expression patterns may help better understand the epigenetic mechanisms underlying tumor heterogeneity in patients with colon cancer and provide more personalized therapeutic strategies.

**Supplementary Information:**

The online version contains supplementary material available at 10.1186/s13148-022-01290-y.

## Background

Colon cancer remains a major source of morbidity and mortality worldwide [1].

Similar to many other malignancies, Colon cancer is also a heterogeneous disease with distinct molecular properties, resulting in diverse clinical outcomes [2, 3]

Although several molecular classification strategies have been proposed to characterize distinct biological properties in colon cancer [3], more effective and clinically accessible classifiers remain to be explored.

Histone modification is an important epigenetic method used to regulate chromatin structure, DNA repair, and gene expression. It plays a crucial role in oncogenic transformation and variations in therapeutic responses [4]. Although many types of histone modification have been reported so far, acetylation and methylation are the two most well-studied types [4], and there are functional interactions between them [5]. Histone acetylation has been recognized as a fundamental process that regulates gene transcriptional activation by neutralizing the positive charge at unmodified lysine residues to diminish the electrostatic affinity between DNA and histones to enable transcription factors to more easily bind to the promoter region [6]. It is also a dynamic and reversible process regulated by two kinds of enzymes with opposite effects: acetyltransferases (acetyl group transfer onto lysine residues) and deacetylases (acetyl group removal from lysine residues). Similarly, histone methylation is also tightly controlled by several methyltransferases (methyl group transfer onto lysine residues) and demethylase enzymes (methyl group removal from lysine residues) that function in concert to transfer and remove specific methyl groups critical for gene expression, cell fate, and genomic stability [7, 8]. However, compared with acetylation, histone methylation is more complex and subtle and is considered to be the most stable and inheritable chromatin modification form of all histone modifications [7, 8].

It has been widely reported that alterations in histone acetylation and methylation patterns and their interactions are linked with the initiation and progression of colon cancer [9–11]. However, most studies were conducted on one or two histone modification regulators due to technical limitations. The global effect of these regulators on biological outcomes and whether their interactions help classify patients from the perspective of histone modification in colon cancer remains unclear. Therefore, in this study, we comprehensively evaluated histone modifier expression patterns by clustering the transcriptomic expressions of 88 histone acetylation and methylation regulators in an unsupervised manner in an integrated cohort comprising 1372 patients with colon cancer from The Cancer Genome Atlas (TCGA) and Gene Expression Omnibus (GEO) databases. Moreover, we established a scoring scheme capable of individually quantifying histone modification status and predicting the clinical outcomes and fluorouracil (the basic drug of adjuvant chemotherapy for colon cancer) responses, designated as “HM_score.” Moreover, by performing a genome-wide screening of the “Clustered Regularly Interspaced Short Palindromic Repeats (CRISPR)” library, we demonstrated that ZEB2 acts as a driver gene mediating the fluorouracil resistance related to histone modifier expression patterns.

## Results

### Landscape of the genetic variation of histone modification regulators in colon cancer

**Fig. 1:**
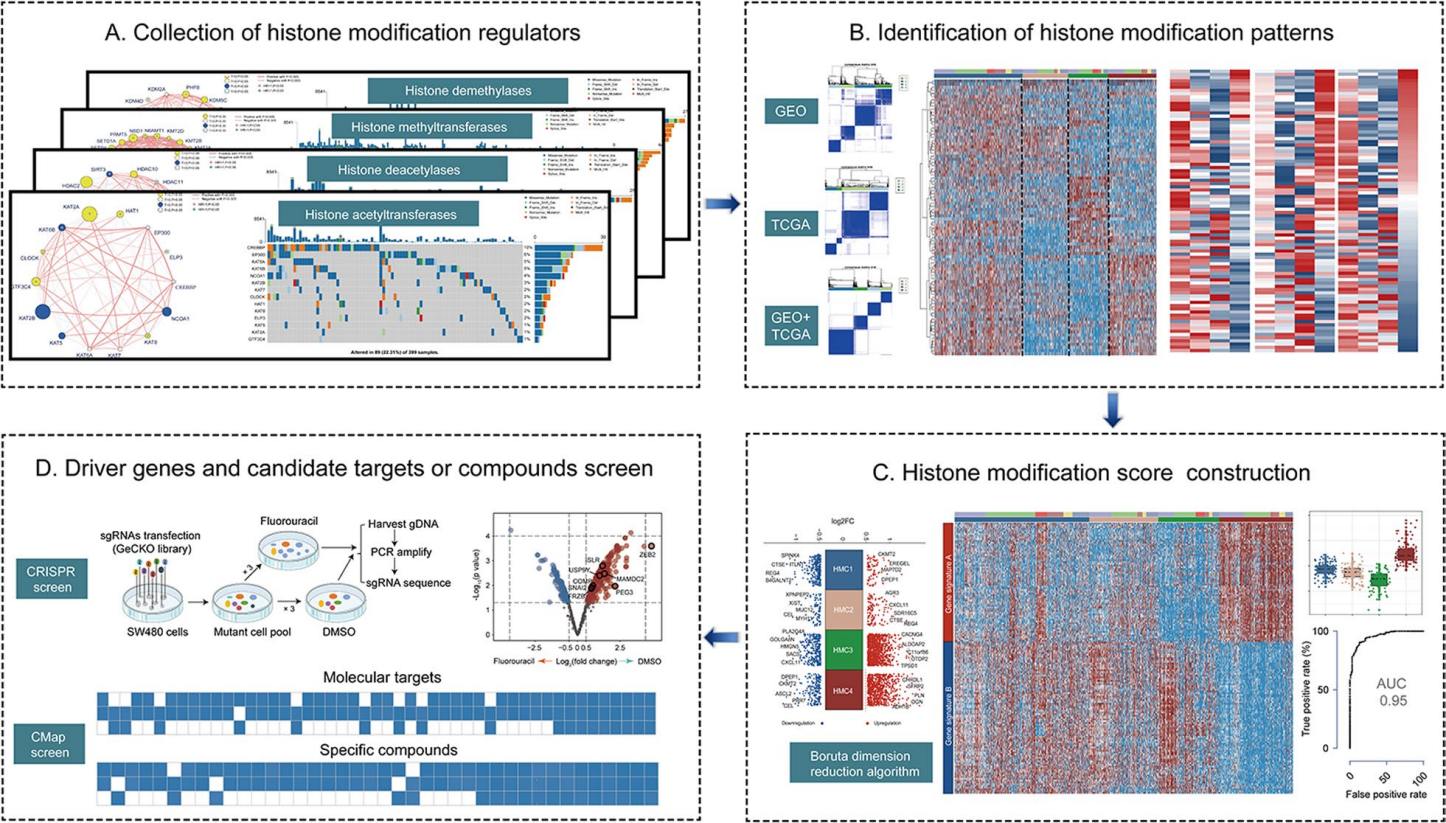
Workflow diagram of this study. The main steps conducted in the study were as follows: (1) the collection of histone modification regulators by searching published articles; (2) the identification of histone modifier expression patterns using an unsupervised clustering analysis; (3) the construction of the histone modification score by the Boruta dimension reduction algorithm; and (4) the identification of the driver genes mediating the fluorouracil resistance related to histone modifier expression patterns and the candidate targets or compounds for the chemosensitization of patients in the HMC4 cluster by conducting a genome-wide screening of the CRISPR-Cas9 library and a Connectivity Map analysis, respectively

**Fig. 2:**
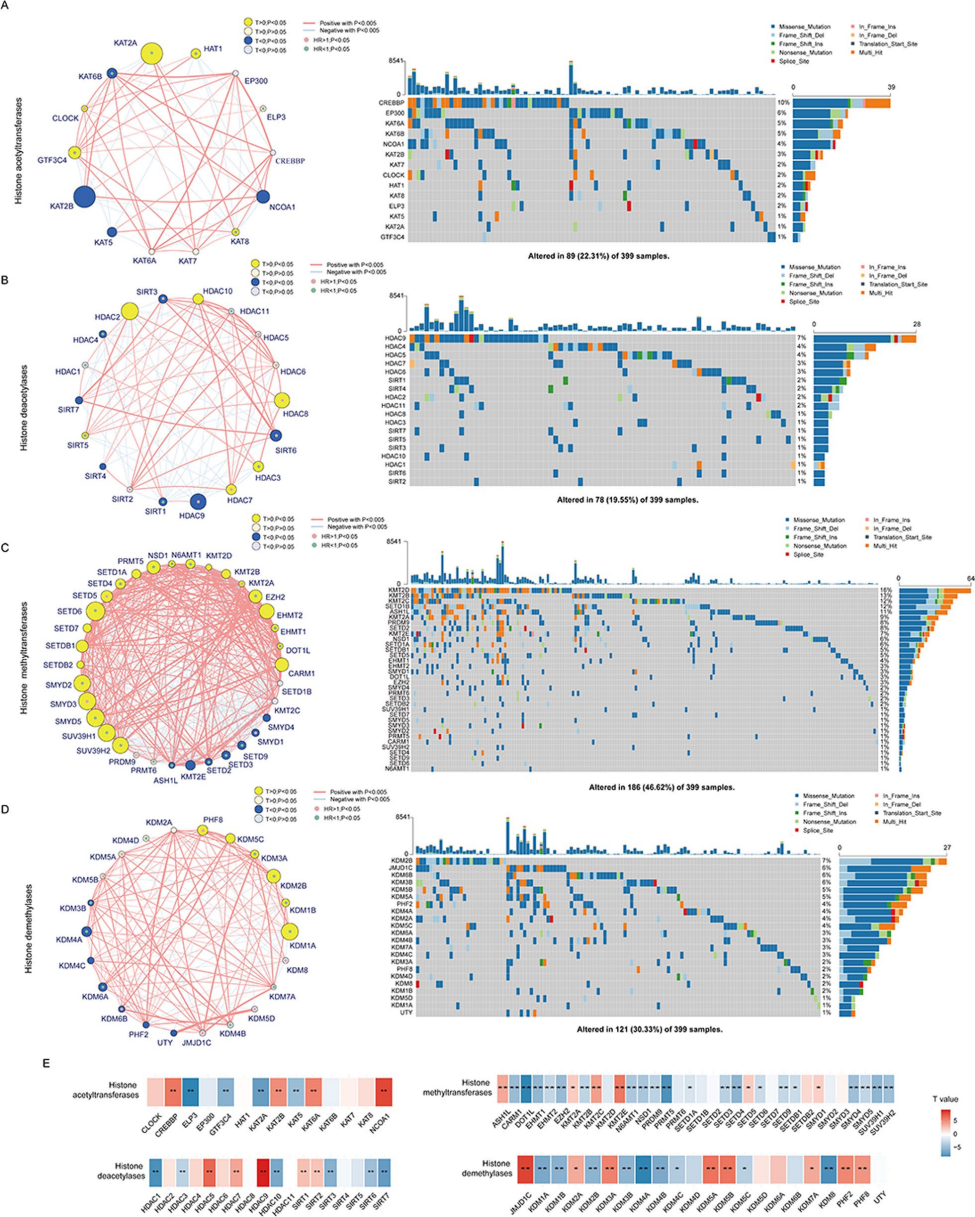
Landscape of the transcriptomic and genetic alterations of histone modification regulators in colon cancer. **A**–**D** (left) Correlations, expressions, and prognosis of histone acetyltransferases (**A**, left), histone deacetylases (**B**, left), histone methyltransferases (**C**, left), and histone demethylase regulators (**D**, left) in patients with colon cancer. The red line represents a positive correlation with a *p* < 0.00001, and the blue line represents a negative correlation with a *p* < 0.00001. Yellow circles indicate a higher gene expression in colon cancer than in normal colon tissue. Blue circles indicate a lower gene expression in colon cancer than in normal colon tissue. Circle size represents the absolute value of the t-statistics obtained from the Student’s t test. The green points inside circles represent favorable factors for relapse-free survival, and the red points represent risk factors for relapse-free survival. **A**–**D** (right) The mutation frequency of histone acetyltransferases (**A**, right), histone deacetylases (**B**, right), histone methyltransferases (**C**, right), and histone demethylases regulators (**D**, right) in the TCGA-COAD cohorts. Each figure column represents one patient. The upper bar plot represents the total tumor mutation burden of patients. The number on the right shows the mutation frequency of each regulator. The right bar plot indicates the proportion of each variant type. **E** Matrix heatmap of the differential expression of histone modification regulators between the fluorouracil-response and fluorouracil-nonresponse groups. The expression of each histone modification regulator was compared by means of a two-sided Student’s t test. Red marked squares indicate higher expression in the fluorouracil-nonresponse group than in the fluorouracil-response group, and blue marked squares indicate higher expression in the fluorouracil-response group than in the fluorouracil-nonresponse group. *p < 0.05 and **p < 0.01

Figure 1 represents the work flow of this study. We retrieved four reviews on histone acetylation and methylation modification [4, 7, 8, 12], and a total of 88 acknowledged histone modification regulators, including 14 acetyltransferases, 18 deacetylases, 34 methyltransferases, and 22 demethylases, were identified and summarized for subsequent analysis (Additional file 1: Table S1). To clarify the role of histone modification regulators in patients with colon cancer, the gene expression profile of 884 samples of colon tumor and 60 samples of nonneoplastic mucosa were collected from the GSE39582 and TCGA-COAD datasets. The comprehensive landscape of the expression pattern, prognostic significance, and interactions between these modifiers were depicted in the network plot (Fig. 2A–D, left). Most regulators demonstrated significant differential transcriptional expression between tumor and normal tissues and were significantly correlated with relapse-free survival (RFS, Additional file 1: Table S2), indicating that histone modification may play a crucial role in the pathogenesis and progression of colon cancer. As for somatic mutations, we found that 237 of the 399 included samples (59.4%) demonstrated at least one altered histone modification regulator (defined as the total mutation rate); nevertheless, the mutation rate of most regulators was less than 10% (Fig. 2A–D, right). Interestingly, we noticed that the four regulators with the highest mutation frequency in colon cancer, namely KMT2D (64/399, 16.0%), KMT2B (52/399, 13.0%), KMT2C (48/399, 12.0%), and SETD1B (46/399, 11.5%), were all histone H3 lysine 4 (H3K4) methyltransferases that belonged to the “Complex of Proteins Associated with Set1” family [13]. The landscape of the mutation positions of these genes is displayed in Additional file 2: Fig. S1A–D. Furthermore, tumor microenvironment (TME) analyses revealed significantly higher immune cell infiltration abundance, especially for cytotoxic cells, CD8^+^ T cells, activated dendritic cells, and Th2 cells, in samples from patients with mutations in any one of KMT2D, KMT2B, KMT2C, or SETD1B than in samples from patients without these mutations (Additional file 2: Fig. S1E). Consistently, the Sankey plot (Additional file 2: Fig. S1F) also showed that the mutations of these four “Complex of Proteins Associated with Set1” family genes were mainly concentrated in patients with the high microsatellite instability (MSI-H), CMS1(consensus molecular subtypes) [14], C2 (pan-immune, TCGA) [15], or HM-indel (pan-GI, TCGA) [15] colon cancer subtypes, which mostly indicate immune activation phenotypes. We also studied the prevalence of histone modifier gene alterations across tumor types (Additional file 3: Fig. S2A–D and Additional file 1: Table S3). The total mutation rate of histone modifiers in colon cancer samples (59.4%) was slightly lower than the average (64.7%) and was only higher than that in the liver (47.3%) and breast cancer (39.1%) samples. Interestingly, besides colon cancer, KMT2D also demonstrated a relatively high alteration frequency (> 10%) in most other tumor types. However, SETD1B mutation was only concentrated in colon cancer samples and was not detected in most other tumor types. Finally, we explored the potential role of histone modification regulators in regulating chemoresponses by comparing the expression of each histone modification regulator between fluorouracil response and nonresponse groups using a two-sided Student’s t test in the dataset merged by GSE39582 and TCGA-COAD. As shown in Fig. 2E, most regulators presented significant differential expressions among the nonresponse and response subgroups of fluorouracil, indicating that these regulators may affect the efficacy of adjuvant chemotherapy in patients with colon cancer. Collectively, the above results indicated that expression alterations and genetic variations in histone modifiers were important factors contributing to tumor heterogeneity and were closely linked with the initiation, progression, and therapeutic effect of colon cancer.

### Identification of histone modifier expression patterns and exploration of their clinical relevance

**Fig. 3:**
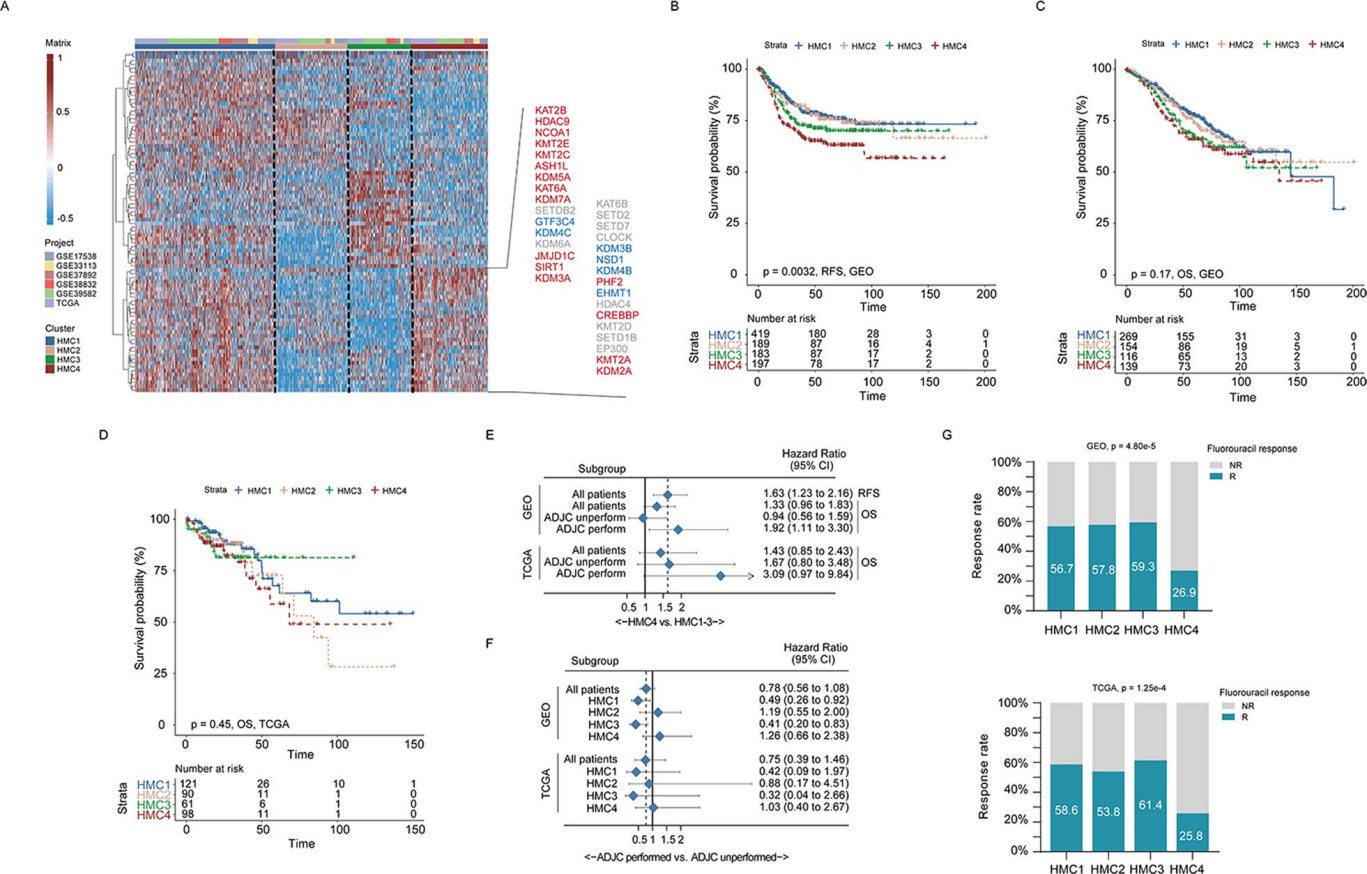
Consensus clustering of histone modification regulators in patients with colon cancer. **A** Heatmaps demonstrating the histone modifier expression patterns in patients with colon cancer identified by the unsupervised clustering analysis of 88 histone modification regulators in the integrated meta-GEO and TCGA-COAD cohort. Cohort details and histone modification clusters are used as patient annotations. We specifically labeled the names of histone modification regulators highly expressed in patients in the HMC4 cluster. Red values indicate significantly higher expression in the fluorouracil-nonresponse group than in the fluorouracil-response group, blue values indicate significantly higher expression in the fluorouracil-response group than in the fluorouracil-nonresponse group, and gray values indicate statistically insignificant differences. **B** and **C** Kaplan–Meier curves of relapse-free survival (**B**) and overall survival (**C**) according to histone modifier expression patterns in the meta-GEO cohort. **D** Kaplan–Meier curves of overall survival according to histone modifier expression patterns in the TCGA-COAD cohort. **E** Forest plots of the association between HMC4 pattern and overall survival in subgroups stratified by adjuvant chemotherapy conduction. **F** Forest plots of benefits of adjuvant chemotherapy in different HMC clusters in the GSE39582 and TCGA-COAD cohorts. Unadjusted hazard ratios (boxes) and 95% confidence intervals (horizontal lines) are depicted; **G** bar charts summarize the proportions of patients with fluorouracil-response signatures and those with nonresponse signatures within and across different histone modifier expression patterns. RFS, relapse-free survival; OS, overall survival; CI, confidence interval; ADJC, adjuvant chemotherapy; R, response; and NR, nonresponse

As histone modifications were reported to play a crucial role in the tumorigenesis and progression of colon cancer by causing abnormal epigenomic reprogramming [4], we aimed to evaluate whether the transcriptional profiling of the 88 acetylation and methylation regulators can help classify patients with colon cancer. A total of 1372 patients diagnosed with stage I–III colon cancer from 5 GEO datasets (GSE17536, GSE33113, GSE37892, GSE38832, and GSE39582) and the TCGA-COAD dataset were enrolled (Additional file 1: Tables S4, S5). Unsupervised K-means clustering analyses of the meta-GEO cohort (990 patients), TCGA-COAD cohort (382 patients), and integrated meta-GEO and TCGA-COAD cohort (1372 patients) were conducted. Results have provided four distinct expression patterns of histone modifiers (Additional file 4: Fig. S3A–C), and the compositions of histone modifiers in these clusters were similar among all three cohorts (Additional file 4: Fig. S3D), indicating that the existence of these four clusters was stable. We termed these clusters HMC1 (*n* = 546), HMC2 (*n* = 280), HMC3 (*n* = 247), and HMC4 (*n* = 299). Among them, HMC1 exhibited a high expression abundance of nearly all histone modifiers, indicating that the activity and turnover of histone acetylation and methylation were intense in HMC1, while the remaining three clusters showed the enrichment of partial regulators (Fig. 3A). The distribution of each histone modifiers in the four clusters is shown in Additional file 4: Fig. S3E, F. Specifically, we noticed that HMC4 was characterized by the prominent expression of regulators enriched in the fluorouracil-nonresponse subgroup (Fig. 3A). A survival analysis revealed that HMC4 had a significantly shorter RFS time than did HMC1, HMC2, and HMC3 (HMC4 vs. HMC1–3: hazard radio [HR] = 1.63, 95% confidence interval [CI] 1.23–2.16, Fig. 3B). As for overall survival (OS), HMC4 also exhibited a higher mortality risk than the remaining clusters; however, this difference was statistically insignificant (Fig. 3C–E). It should be noted that the negative correlation between HMC4 and OS obviously increased in patients who underwent adjuvant chemotherapy (GSE39582: HR 1.92, 95%CI 1.11–3.30; TCGA-COAD: HR 3.09, 95%CI 0.97–9.84), suggesting that this pattern may be associated with chemotherapy resistance (Fig. 3E). To validate this hypothesis, we analyzed the relationship between HMC clusters and chemotherapy responses in both GSE39582 and TCGA-COAD datasets. As shown in Fig. 3F–G, adjuvant chemotherapy conduction did not provide any survival benefits to patients in the HMC4 cluster in both GSE39582 (HR 1.26, 95%CI 0.66–2.38) and TCGA-COAD (HR 1.03, 95%CI 0.40–2.67) cohorts and the fluorouracil-response rate was also the lowest in patients in the HMC4 cluster. Taken together, these data imply that the histone modification clusters were significantly correlated with patients’ prognosis and chemotherapy benefit, which might provide new insights on colon cancer classification system.

### Biological characteristics of different histone modifier expression patterns

**Fig. 4:**
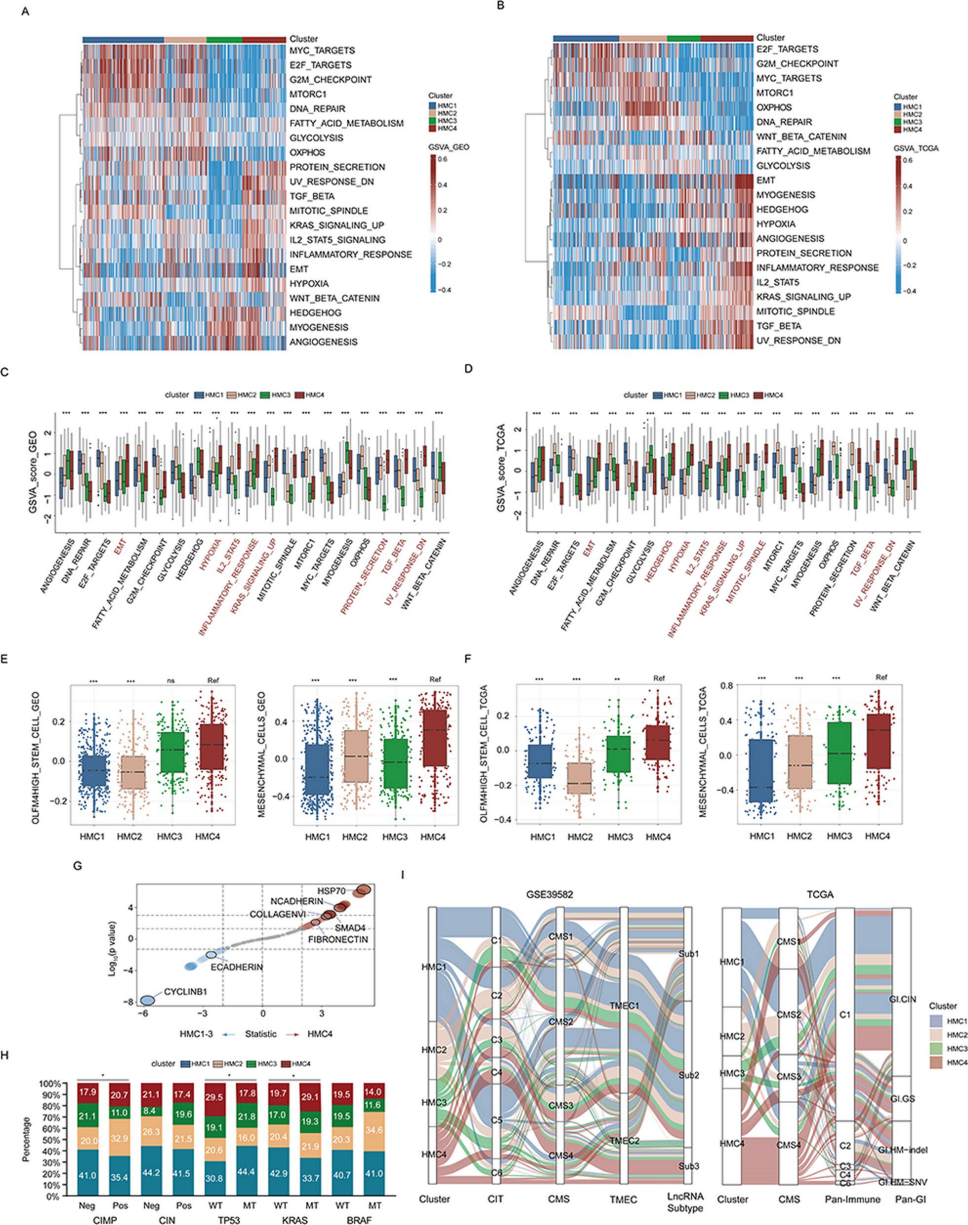
Biological function characteristics of distinct histone modifier expression patterns. **A** and **B** Heatmaps show the results of the gene set variation analysis (GSVA) based on “hallmark gene sets” in the four identified histone modifier expression patterns in the meta-GEO (**A**) and TCGA-COAD (**B**) cohorts. Red values represent activated pathways, and blue values represent inhibited pathways. Histone modifier expression patterns are used as sample annotations. **C** and **D** Boxplots of GSVA results based on “hallmark gene sets” in the four studied histone modifier expression patterns in the meta-GEO (**C**) and TCGA-COAD (**D**) cohorts. Boxes represent 25–75% of values, lines in boxes represent median values, whiskers represent 1.5 interquartile ranges, and black dots represent outliers. Red terms indicate that the corresponding pathway has the highest activation level in patients. **E** and **F** Boxplots of OLFM4 high stem cell abundance (left) and mesenchymal cell abundance (right) in the four studied histone modifier expression patterns in the meta-GEO (**E**) and TCGA-COAD (**F**) cohorts. Boxes represent 25–75% of values, lines in boxes represent median values, whiskers represent 1.5 interquartile ranges, and black dots represent outliers. **G** Scatter plots represent the comparison of the protein expression level of the pathway marker genes between patients in the HMC1-3 and HMC4 clusters. **H** Bar charts summarize the proportions of histone modifier expression patterns in and across different molecular characteristic subgroups. **I** Sankey diagram of histone modification clusters in groups with different molecular subtypes in the GSE39582 (left) and TCGA-COAD (right) cohorts. *p < 0.05, **p < 0.01, ***p < 0.001; ns, not significant; Ref, reference; CIMP, CpG island methylator phenotype; CIN, chromosome instability; MT, mutant type; WT, wild type; CMS, consensus molecular subtypes; and TMEC, tumor microenvironment cluster

To further characterize and understand the biological differences between these intrinsic histone modification phenotypes, we performed a gene set variation analysis (GSVA) based on the “Hallmarker” gene set (Fig. 4A–D). Results indicated that there are some similarities in the biological pathway activation between HMC1 and HMC2. For example, the activation levels of DNA repair-, E2F-, mTORC1-, and MYC-related pathways in HMC1 and HMC2 samples were significantly higher than those in HMC3 and HMC4. Interestingly, the G2M checkpoint pathway score was the highest in HMC1 but the lowest in HMC3 in both the meta-GEO and TCGA-COAD cohorts, suggesting that cell cycle disorder may be an important mechanism underlying the tumorigenesis of patients with HMC1. In addition, the protein secretion pathway was also significantly inhibited in HMC3 patients. In both meta-GEO and TCGA-COAD cohorts, HMC2 was enriched in multiple cell metabolism pathways, including glycolysis, fatty acid metabolism, and oxidative phosphorylation, suggesting that inhibition of metabolism may be a potential treatment strategy for HMC2 patients. Meanwhile, the Wnt signaling pathway and Hedgehog pathway, two key signaling pathways that are crucial for stem and progenitor cell homeostasis and function, were lowest in HMC2 patients, suggesting that the stemness feature of HMC2 is weaker than that of other histone modifier expression patterns. Particularly, HMC4 represented a stromal/mesenchymal phenotype with many enriched pathways, including epithelial–mesenchymal transition (EMT), hypoxia, and TGFβ signaling. Consistent with the results of GSVA, the protein expression levels of the molecular markers involved in EMT and TGFβ signaling were significantly higher in HMC4 than in the remaining clusters (Fig. 4G). Intriguingly, we found that the KRAS pathway showed the highest activation degree in HMC4, and the number of patients in the HMC4 cluster with KRAS mutant tumors was significantly higher than those with KRAS wild-type tumors (Fig. 4H). We further calculated the level of OLFM4^+^ stem cells and mesenchymal cells using signatures obtained from single-cell sequences proposed by Gao et al. [16] and analyzed their associations with histone modifier expression patterns. Results have confirmed that the stem cell and mesenchymal cell signatures both had the highest enrichment in patients of the HMC4 cluster (Fig. 4E, F). Finally, based on the molecular subtypes of the GSE39582 and TCGA-COAD cohorts (Fig. 4I), we found that most patients with the C4 (CIT) [17], C6 (CIT) [17], CMS4, TMEC2 [18], Sub 3 [19], and C6 (Pan-Immune, TCGA) [20] subtypes, which mostly represent stromal/mesenchymal phenotypes, were assigned to the HMC4 cluster. Overall, these results suggest that histone modifier expression patterns were characterized by distinct biological pathway activation status.

### Immune landscapes of different histone modifier expression patterns

**Fig. 5:**
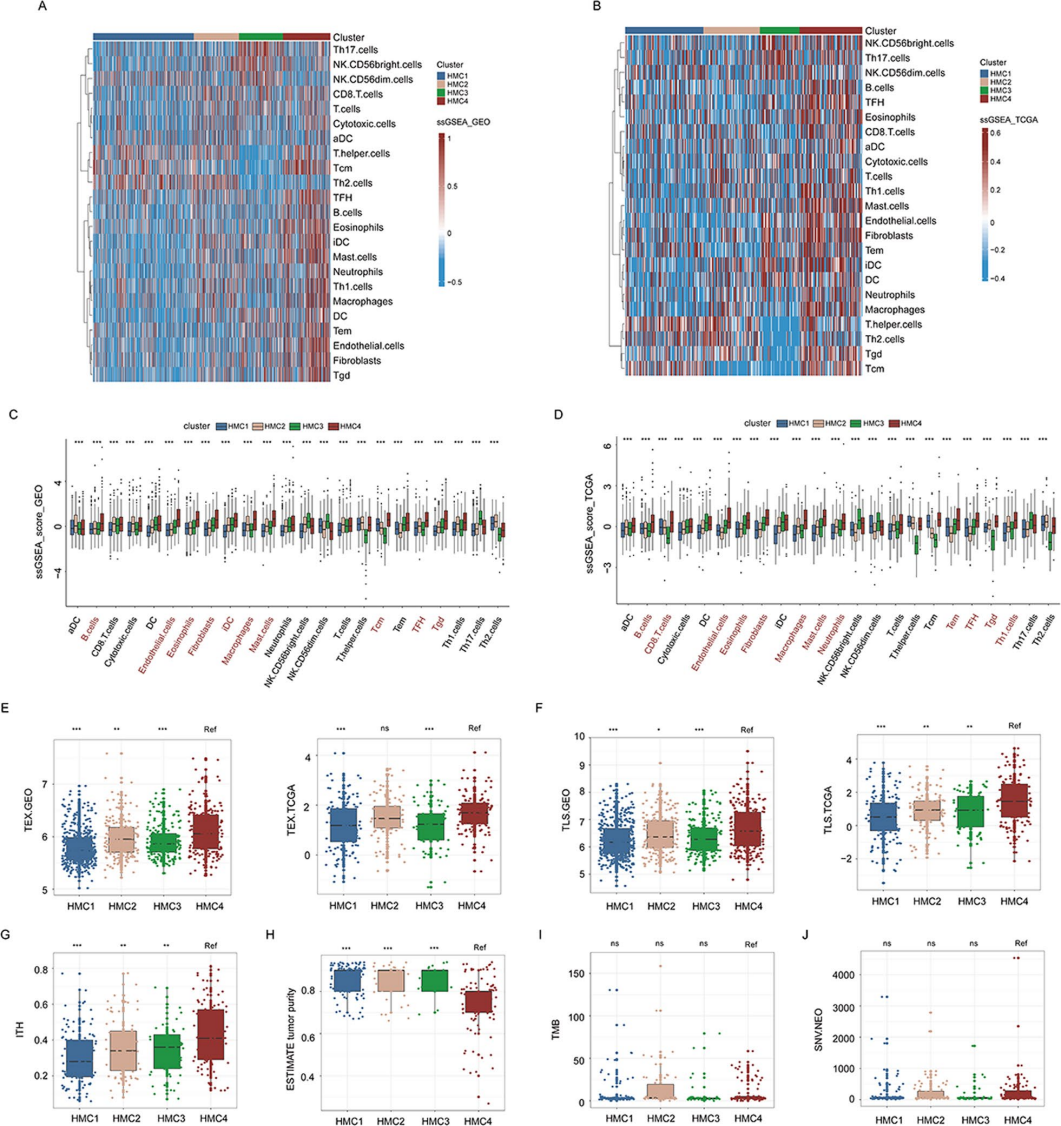
Tumor microenvironment characteristics of distinct histone modifier expression patterns. **A** and **B** Heatmaps show immune cell infiltration in the four studied histone modifier expression patterns in the meta-GEO (**A**) and TCGA-COAD (**B**) cohorts. Red values represent highly infiltrated cells, and blue values represent minimally infiltrated cells. Histone modifier expression patterns are used as sample annotations. **C** and **D** Boxplot of immune cell infiltration in the four studied histone modifier expression patterns in the meta-GEO (**C**) and TCGA-COAD (**D**) cohorts. Boxes represent 25–75% of values, lines in boxes represent median values, whiskers represent 1.5 interquartile ranges, and black dots represent outliers. Red terms indicate the highest level of infiltration in patients in the HMC4 cluster. **E** and **F** Boxplot of T cell exhaustion level (**E**) and tertiary lymphoid structure signatures (**F**) in the four studied histone modifier expression patterns in the meta-GEO (left) and TCGA-COAD (right) cohorts. Boxes represent 25–75% of values, lines in boxes represent median values, whiskers represent 1.5 interquartile ranges, and black dots represent outliers. **G**–**J** Boxplot of intratumoral heterogeneity levels (**G**), tumor purity (**H**), tumor mutation burden (**I**), and neoantigen (**J**) in the four studied histone modifier expression patterns in the TCGA-COAD cohort. Boxes represent 25–75% of values, lines in boxes represent median values, whiskers represent 1.5 interquartile ranges, and black dots represent outliers. *p < 0.05, **p < 0.01, ***p < 0.001; ns, not significant; TEX, T cell exhaustion; TLS, tertiary lymphoid structure; ITH, intratumoral heterogeneity; TMB, tumor mutation burden; single-nucleotide variant; and NEO, neoantigen

We subsequently explored differences in the immune landscapes among all histone modification clusters. A single-sample gene set enrichment analysis (ssGSEA) was performed to obtain the infiltration abundance of TME cells as described in the “Methods” section. As shown in Fig. 5A–D, the TME features of patients in the HMC1 cluster were close to those of an “immune-desert” phenotype characterized by little immune cell infiltration; nevertheless, both HMC2 and HMC3 displayed moderate immune infiltration. However, helper T cells, especially Th2 cells, and central memory T cells were markedly downregulated in HMC3 compared with those in the remaining clusters, suggesting that antigen recognition was repressed in HMC3. Compared with the remaining clusters, HMC4 was characterized by significant increases in stromal cell infiltration, such as fibroblasts and endothelial cells. Moreover, innate immune cells with immunosuppressive properties, such as macrophages, neutrophils, mast cells, and B cells, also had the highest infiltration rate in HMC4. It is noteworthy that we found that CD8^+^ T cells, which are considered marker cells of adaptive immunity, were more abundant in HMC4 than in the remaining clusters in the TCGA-COAD cohort. Previous studies have revealed that the immune-excluded TME phenotype was characterized by an abundance of immune cells, with these immune cells being retained in the stroma surrounding tumor cell nests rather than in the parenchyma [21]. Therefore, we speculated that the TME feature of patients in the HMC4 cluster might be classified as the feature of the “immune-excluded” phenotype. Subsequent analyses have revealed that patients in the HMC4 cluster had the highest T cell exhaustion [22], tertiary lymphoid structure signatures [23], and stromal cell infiltration intensity score [24] (Fig. 5E, F, and Additional file 5: Fig. S4). Moreover, we obtained intratumoral heterogeneity (ITH), tumor purity, tumor mutation burden (TMB), and the number of neoantigen results from the study of Thorsson et al. [15] and analyzed their distribution across histone modifier expression patterns. Consistent with our earlier findings, patients in the HMC4 cluster exhibited the highest ITH and lowest tumor purity (Fig. 5G, H). However, there were no significant differences in TMB and number of neoantigen among HMC clusters (Fig. 5I, J). In summary, these data proved that the HMC4 cluster was closely related to the “immune-excluded” phenotype that was characterized by enrichment of both immune and stromal cell types.

### Histone modification score (HM_score) construction

**Fig. 6:**
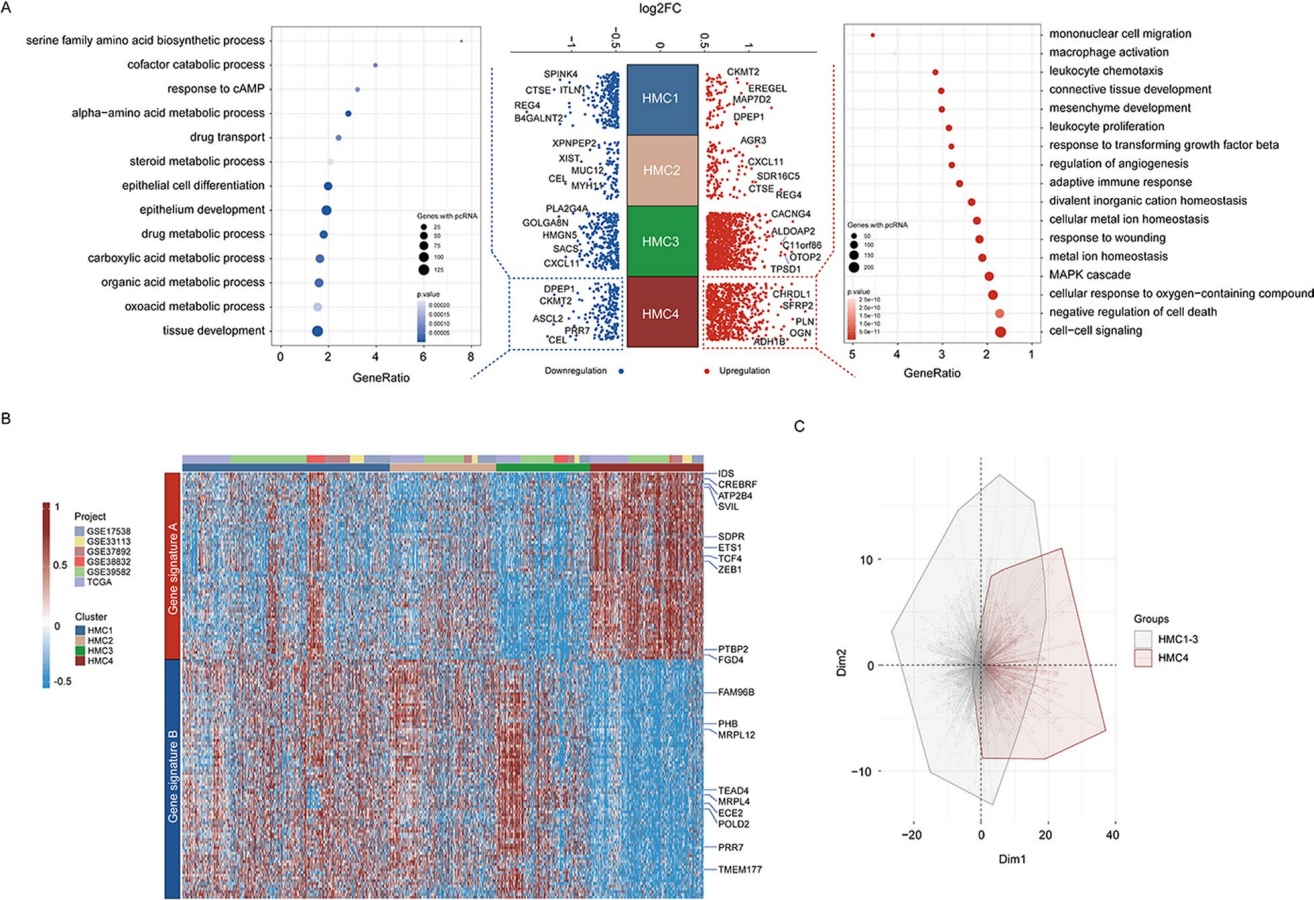
Construction of the histone modification score. **A** Differential gene expression analysis showing up (red)- and downregulated genes (blue) in all four histone modification clusters (middle). A gene ontology analysis depicted the enriched pathways of the genes downregulated (left) and upregulated (right) in patients in the HMC4 cluster. Circle size represents the number of genes enriched in this pathway. Circle color depth indicates *p* value. **B** Heatmap shows the gene expression patterns of histone modification clusters after dimension reduction using the Boruta algorithm. Red values represent high expression, and blue values represent low expression. Cohort details and histone modification clusters are used as patient annotations. **C** Principal component analysis of differentially expressed genes to distinguish HMC4 from other histone modifier expression patterns. FC, fold change

Since patients in the HMC4 cluster had the worst prognosis and lowest fluorouracil-response rate, we believe that developing a scoring model capable of individually quantifying histone modification status to identify patients in the HMC4 cluster may offer potential clinical application value. Therefore, we recognized differentially expressed genes (DEGs) in these four clusters. A total of 1003 DEGs (801 upregulated and 202 downregulated) in HMC4 were identified (Fig. 6A and Additional file 1: Table S6). A gene ontology analysis of these DEGs revealed that the upregulated genes were enriched in biological processes related to mesenchyme development, stromal activation, and cell response to external stress, whereas the downregulated DEGs were enriched in items related to cell metabolic processes (Fig. 6A and Additional file 1: Table S7). Subsequently, the Boruta algorithm was applied to reduce the dimension of these DEGs (Methods section), and we ultimately screened out 155 genes to form a histone modification-related signature termed as the HM_score (Fig. 6B, C and Additional file 1: Table S8). The boxplots (Additional file 6: Fig. S5A, B, left) have shown that the median HM_score value was highest in the HMC4 cluster in both meta-GEO and TCGA-COAD cohorts. A receiver operating characteristic (ROC) curve analysis further demonstrated that HM_score was a reliable index to distinguish patients in the HMC4 cluster with an area under the ROC curve (AUC) of 0.94 and 0.95 in the meta-GEO dataset and in the TCGA-COAD dataset, respectively (Additional file 5: Fig. S4A, B, right). In conclusion, the above results strongly suggested that the HM_score can effectively distinguish HMC4 patients.

### Clinical relevance and biological characteristics of HM_score

**Fig. 7:**
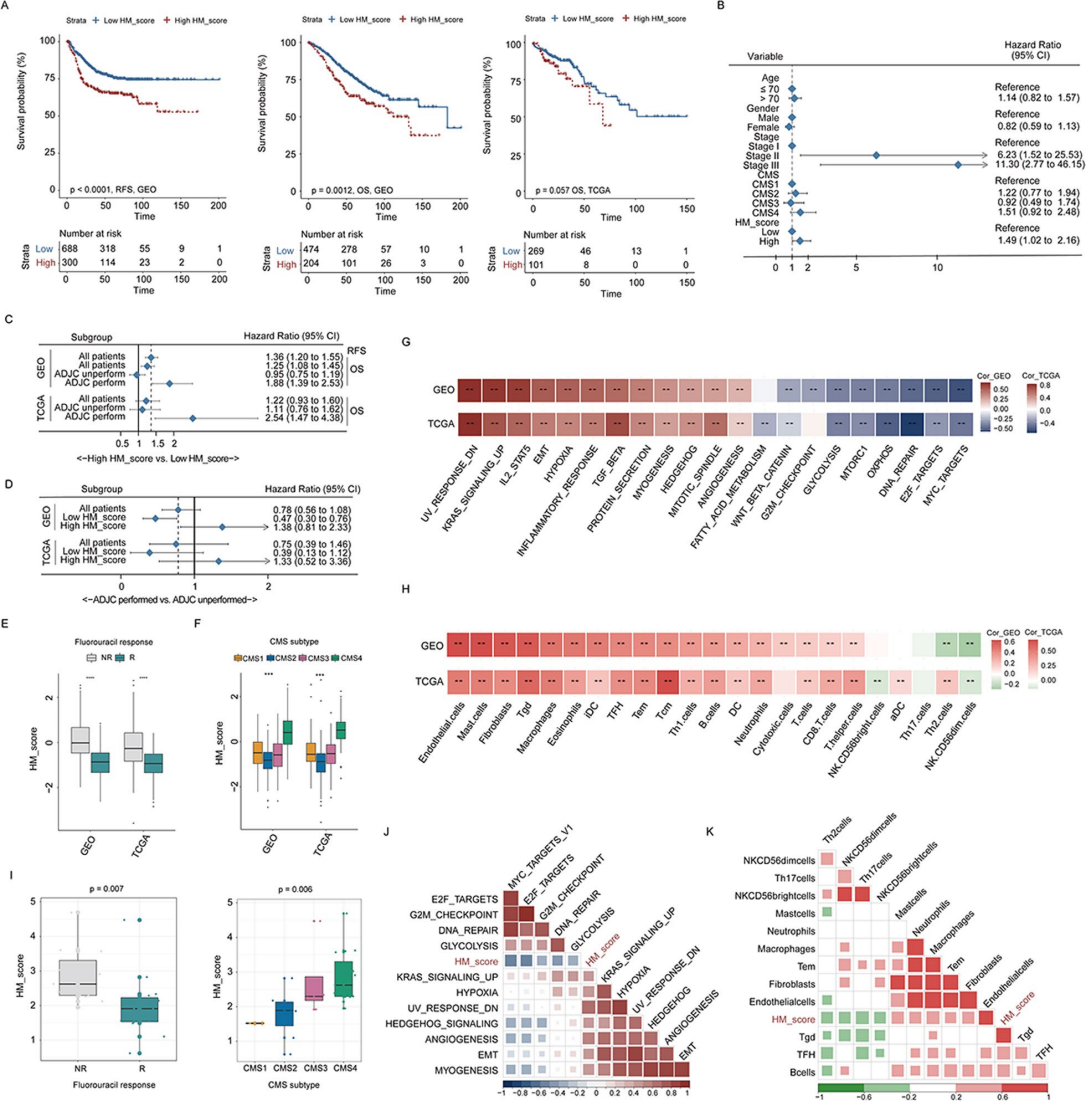
Clinical significance and biological function of the histone modification score. **A** Kaplan–Meier curves of relapse-free survival (left) and overall survival (middle) in the meta-GEO cohort and of overall survival (right) in the TCGA-COAD cohort according to the HM_score. **B**–**D** Forest plots of the association between the HM_score value and relapse-free survival in a multivariate Cox analysis (**B**); forest plots of the association between HM_score value and overall survival in subgroups stratified by adjuvant chemotherapy conduction (**C**); forest plots of the benefits of adjuvant chemotherapy in the low- and high-HM_score groups in the meta-GEO and TCGA-COAD cohorts (**D**). Unadjusted hazard ratios (boxes) and 95% confidence intervals (horizontal lines) are depicted. **E** and **F** Boxplot of HM_score values among patients with different fluorouracil responses (**E**) and among patients with different CMS molecular subgroups (**F**) in the meta-GEO and TCGA-COAD cohorts. Boxes represent 25–75% of values, lines in boxes represent median values, whiskers represent 1.5 interquartile ranges, and black dots represent outliers. **G** and **H** Heatmaps of the correlation between the HM_score value, pathway activation (**G**), and tumor microenvironment cell infiltration (**H**) in the meta-GEO (upper) and TCGA-COAD (down) cohorts. *p < 0.05 and **p < 0.01. **I** Boxplot of the HM_score values among patients with different fluorouracil responses (left) and among patients with different CMS molecular subgroups (right) in the SYSUCC cohort. Boxes represent 25–75% of values, lines in boxes represent median values, whiskers represent 1.5 interquartile ranges, and black dots represent outliers. **J** and **K** Heatmaps of the correlation between HM_score value, pathway activation (**J**), and tumor microenvironment cell infiltration (**K**) in the SYSUCC cohort. RFS, relapse-free survival; OS, overall survival; CI, confidence interval; CMS, consensus molecular subtypes; R, response; NR, nonresponse; and ADJC, adjuvant chemotherapy

We subsequently explored the prognostic impacts and predictive value of therapeutic benefits of the HM_score. Patients were divided into low- or high-score subgroups according to the cutoff values determined by the “survminer” package. The survival analyses indicated that groups with low HM_score had a significantly high RFS (HR 1.77, 95%CI 1.37–2.29) in the meta-GEO cohort (Fig. 7A). Moreover, the HM_score was validated as an independent prognostic biomarker for evaluating patient relapse using a multivariate Cox regression model (HR 1.49, 95%CI 1.02–2.16, Fig. 7B) controlled for age, gender, tumor stage, and CMS subtype. Similarly, we also noticed that the positive correlation between HM_score value and mortality rate was statistically significant in the subgroup of patients receiving chemotherapy in both the GSE39582 (HR 1.88, 95%CI 1.39–2.53) and TCGA-COAD (HR 2.54, 95%CI 1.47–4.38) cohorts (Fig. 7C), while chemotherapy conduction was a risk factor for unfavorable prognosis in patients within a high HM_score group (GSE39582: HR 1.38, 95%CI 0.81–2.33; TCGA-COAD: HR 1.33, 95%CI 0.52–3.36; Fig. 7D). The following boxplots also showed that HM_score was significantly higher in the fluorouracil-nonresponse and CMS4 subtype groups than in the remaining groups (Fig. 7E, F). GSVA and immune analyses demonstrated that the HM_score was markedly positively correlated with stromal activation processes and stromal cell infiltration, which is consistent with the results of the HMC4 cluster analysis (Fig. 7G, H). To confirm the clinical value and biological implication of the HM_score, we obtained bulk RNA-sequencing data from 30 additional patients with colon cancer from the Sun Yat-sen University Cancer Center (SYSUCC) as an external dataset (Additional file 1: Table S5). Patients were also grouped into the HMC4 and non-HMC4 using the nearest template prediction algorithm (GenePattern module “NTP,” https://cloud.genepattern.org) based on the DEGs in HMC4 we earlier identified. Consistent with the results of the meta-GEO and TCGA-COAD databases, the median HM_score value was significantly higher in the HMC4 than in the non-HMC4 cluster in the SYSUCC cohort (Additional file 5: Fig. S4C, left), and HM_score defined the HMC4 patterns with an AUC of 0.98 according to ROC curve analysis (Additional file 6: Fig. S5C, right). Furthermore, HM_score was also significantly higher in the fluorouracil-nonresponse and CMS4 subtype groups than in other groups (Fig. 7I), and there were strong positive associations between HM_score and stroma-relevant signatures (Fig. 7J) and stroma cell infiltration (Fig. 7K) in the SYSUCC cohort. The above results revealed that HM_score was a useful biomarker that could effectively predict survival and chemotherapy benefit in colon cancer patients and may offer potential clinical application value.

### Whole-genome CRISPR screen reveals ZEB2 as the candidate driver gene for fluorouracil resistance in patients within HMC4 cluster

**Fig. 8:**
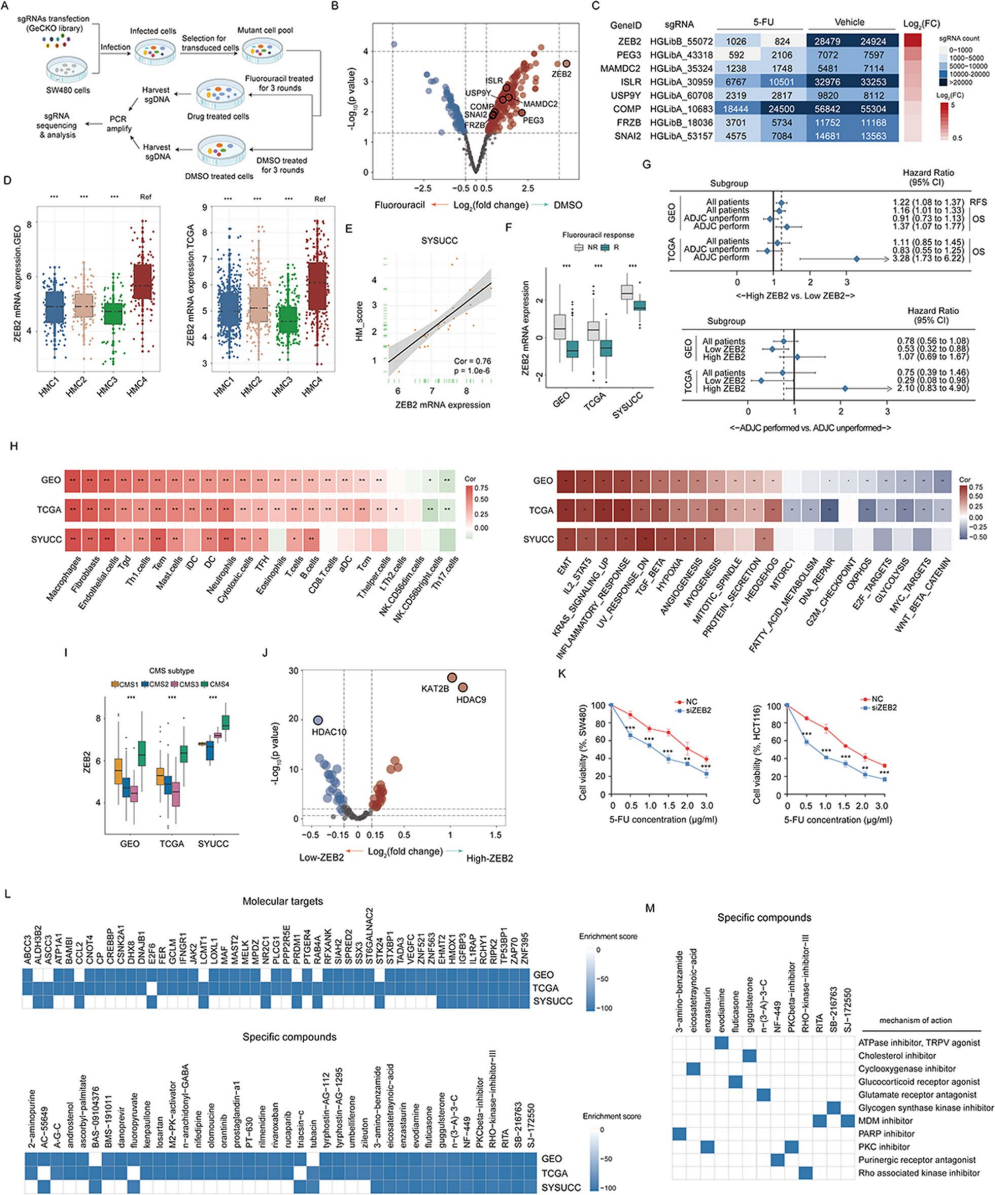
Screening of driver genes and candidate targets or compounds. **A** Experimental outline of screening and analysis. **B** Volcano plots to compare differences in sgRNA abundance between fluorouracil- and vehicle-treated cells. **C** Heatmap showing the counts of sgRNAs representing genes mediating fluorouracil resistance in patients in the HMC4 cluster. **D** Boxplot of ZEB2 expression in the four studied histone modifier expression patterns in the meta-GEO (left) and TCGA-COAD (right) cohorts. **E** Scatter plots show the correlation between ZEB2 expression and HM_score value in the SYSUCC cohort. **F** Boxplot of ZEB2 expression among patients with different fluorouracil responses in the meta-GEO, TCGA-COAD, and SYSUCC cohorts. **G** Forest plots of the association between ZEB2 expression and overall survival in subgroups stratified by adjuvant chemotherapy conduction (upper) and the benefit of adjuvant chemotherapy in the low- and high-ZEB2 expression groups in the meta-GEO and TCGA-COAD cohorts (down). **H** Heatmaps of the correlation between ZEB2 expression, tumor microenvironment cell infiltration (left), and pathway activation (right) in the meta-GEO, TCGA-COAD, and SYSUCC cohorts. **I** Boxplot of ZEB2 expression in different CMS molecular subgroups in the meta-GEO, TCGA-COAD, and SYSUCC cohorts. **J** Volcano plots to compare differences in the gene expression of histone modification regulators between the low- and high-ZEB2 expression groups in the integrated meta-GEO and TCGA-COAD cohort. **K** Dose–response curves of SW480 (left) and HCT116 cells (right) transfected with empty vectors or ZEB2 siRNA after fluorouracil treatment for 24 h. The mean ± standard deviation of the three replicates of each time point is shown. **L** Heatmaps showing the enrichment score of each molecular target (upper) and compound (down) in the Connectivity Map analysis. **M** Heatmap showing the mechanisms of the action (rows) of each compound in the Connectivity Map analysis. *p < 0.05, **p < 0.01, ***p < 0.001; RFS, relapse-free survival; OS, overall survival; CI, confidence interval; CMS, consensus molecular subtypes; R, response; NR, nonresponse; ADJC, adjuvant chemotherapy; and FC, fold change

To identify critical genes driving fluorouracil resistance in patients of the HMC4 cluster, we performed CRISPR-based genome-wide loss-of-function screening in SW480 cells, using 2 μg/mL of fluorouracil as an effective selection pressure (Fig. 8A). From this screen, we discovered a subset of sgRNAs targeting 166 genes were significantly enriched in the fluorouracil-treated cells when compared to the vehicle control. These genes were identified as potential drivers for fluorouracil resistance (Fig. 8B). Moreover, among these genes, eight were highly expressed in patients in the HMC4 cluster (Fig. 8B, C). ZEB2, whose sgRNA was decreased the most in fluorouracil-treated populations, gained our attention. Subsequent analyses confirmed that patients in the HMC4 cluster in both the meta-GEO and TCGA-COAD cohorts had the highest ZEB2 mRNA expression distribution (Fig. 8D). There was also a strong positive correlation between ZEB2 transcriptional expression and HM_score in the SYSUCC cohort (Fig. 8E). A clinical relevance analysis has suggested that ZEB2 expression reflected the prognostic value of both RFS and OS, especially in patients who underwent adjuvant chemotherapy (Fig. 8G). The analysis also revealed that only patients in the low-ZEB2 group could significantly benefit from adjuvant chemotherapy (GSE39582: HR 0.53, 95%CI 0.32–0.88; TCGA-COAD: HR 0.29, 95%CI 0.08–0.98, Fig. 8G). The boxplots in Fig. 8F and I showed that ZEB2 mRNA expression was significantly elevated in the fluorouracil-nonresponse and CMS4 subtype groups. Pathway and immune analyses confirmed that the activation level of stroma pathways and stromal cell infiltration significantly increased as ZEB2 expressions increased (Fig. 8H).

To validate the CRISPR/Cas9 library screening results, we transfected siRNAs targeting ZEB2 and the ZEB2 overexpression plasmid in vitro into SW480 and HCT116, respectively, and performed a methyl-thiazolyl-tetrazolium (MTT) assay. As shown in Fig. 8K and Additional file 7: Fig. S6, the cell viability of the ZEB2 silencing group was significantly inhibited, while the cell viability of the ZEB2 overexpression group was significantly enhanced compared with the control group in each fluorouracil concentration gradient we tested. These data suggested that the cytotoxicity of fluorouracil to tumor cells was influenced by the transcriptional abundance of ZEB2. Interestingly, we uncovered several potential histone modification regulators of ZEB2 by differential expression analysis between the high- and low-ZEB2 groups (Fig. 8J), such as HDAC10, HDAC9, and KAT2B, indicating that these regulators might regulate ZEB2 expression in patients with HMC4. Collectively, based on the results from the analysis of these real-world cohorts, we are confident that the ZEB2 found by CRISPR library screening was indeed the core gene mediating chemoresistance in HMC4 patients.

### The Connectivity Map analysis identifies potential molecular targets and compounds capable of reversing transcriptional characteristics in patients with HMC4

To identify candidate molecular targets and compounds that may be options to achieve chemosensitization in patients with HMC4, we analyzed the Connectivity Map project. Briefly, 147 significantly enriched molecular targets (Additional file 1: Table S9) and 91 compounds (Additional file 1: Table S10) were identified, and those identified in at least two cohorts were presented in the heatmap (Fig. 8L). Among these candidate molecular targets, nine were significantly enriched in all three cohorts, and five of them (TP53BP1 [25], RIPK2 [26], EHMT2 [27], IGFBP3 [28], and HMOX1 [29]) have been reported to have cancer- and chemoresistance-promoting activities simultaneously. Particularly, although EHMT2, a member of the histone methyltransferase family, was identified in all three cohorts, the transcription level of EHMT2 was significantly higher in the fluorouracil-response group (Fig. 2E). Of the 91 compounds, 13 were significantly enriched in all three cohorts. A mode-of-action analysis of these 13 compounds revealed 11 shared action mechanisms (Fig. 8M). Additionally, 2 compounds (SJ-172550 and RITA) shared the mode-of-action as the MDM inhibitor (Fig. 8M). The mode-of-action of the PKC inhibitor was also found in two other compounds (Fig. 8M). These findings provided a new perspective for developing effective chemosensitizing treatment strategies in HMC4 patients.

## Discussion

Histone modifications serve as regulatory markers that are essential to control transcription and architecture. Although histone modification deregulation (particularly the well-studied deregulation of acetylation and methylation modifications) has been widely reported to be vital epigenetic mechanisms underlying cancer progression [4], the correlation between the global profiling of histone modification regulator patterns and tumor heterogeneity due to pathway activation or TME infiltration has not been comprehensively recognized.

In this study, by the consensus clustering and analysis of the transcriptome data of 88 histone acetylation and methylation regulators, we, for the first time, identified four distinct different histone modifier expression patterns that were associated with different clinical outcomes, biological pathways, and TME characteristics. Each cluster was enriched by multiple regulators involved in acetylation or methylation processes, implying that these histone modification regulators contribute to the heterogeneous progression of colon cancer in a highly coordinated manner. Among the four clusters provided in this study, HMC4 gained our attention the most as it had the worst prognosis and lowest fluorouracil-response rate. Based on the functional and TME analyses, we observed that patients in the HMC4 cluster had the highest activation levels of EMT, TGFβ signals, and hypoxia pathways and the highest infiltration of stromal cells and immunosuppressive innate immune cells. HMC4 collectively harbored stromal/mesenchymal properties, which can explain the poor prognosis of patients in this cluster. This result is consistent with that of our previous studies, which stated that the stromal pathway activation level is a core determinant of negative chemotherapy outcomes in patients with colon cancer [18, 19]. Intriguingly, patients in the HMC4 cluster demonstrated higher KRAS mutation and KRAS signaling enrichment incidences than patients in other clusters. Since growing evidence has acknowledged the association between KRAS mutation and the adverse prognostic impacts on patients with colon cancer treated with fluorouracil-based chemotherapy [30, 31], we can conclude that the diminished benefits of chemotherapy in patients within the HMC4 cluster also resulted in the combined effects of KRAS mutation and KRAS signaling dysregulation.

Considering the special clinical features of patients in the HMC4 cluster, there is a need to develop a scoring scheme that can individually quantify the histone modification status to distinguish HMC4 from other histone modification subtypes. By applying the dimension reduction method, we successfully established a transcriptome-based quantification system named the “HM_score” to define HMC4 patterns with high accuracy. This finding validated that the abnormal transcriptional activation of oncogenes or, conversely, the repression of tumor suppressors is the main histone modification mechanism underlying tumor heterogeneity progression [4, 32]. Clinical analyses further highlighted that HM_score is an independent prognostic factor for colon cancer and associated with chemotherapeutic responses. This finding also verified our hypothesis that histone modifier expression patterns could be applied in clinical practice to guide therapeutic strategies more precisely for individual patients.

In addition to exploring the implications of the histone modification pattern on colon cancer treatment strategies, we also performed a genome-wide CRISPR screen and identified that ZEB2 is a potential driver gene contributing to the drug resistance in the background of histone modification alterations. ZEB2 is a known EMT regulator whose promoter experiences dynamic histone mark changes during cell transition toward mesenchymal features in response to EMT inducers, such as TGFβ [33–36]. Currently, several histone modification regulators, including DOT1L [34], DNMT1 [36], EZH2 [33], and KDM5B [35], have been reported to play a role in the TGFβ-stimulated ZEB2 transcriptional upregulation of many cancers. To the best of our knowledge, this study is the first to report an association between ZEB2 expression and global histone modifier expression patterns. Future studies to further elucidate the exact mechanism underlying the ZEB2-related histone modification process may be helpful to develop novel cancer therapies. However, PKC activation is involved in histone modification-dependent ZEB2 expression and EMT processes. Additionally, pan-PKC inhibitors suppress EMT by promoting the DNMT1-induced histone methylation of ZEB2 [36]. Coincidentally, through a Connectivity Map analysis, two types of PKC inhibitors were significantly enriched and consequently considered as compounds for the chemosensitization of patients in the HMC4 cluster. Accordingly, systematic preclinical studies investigating the efficiency of PKC inhibitors as combined targeted therapies for patients in the HMC4 cluster are warranted.

Although our study is the first to establish molecular subtypes based on histone modification regulators, providing new insights on the epigenetic mechanisms underlying colon cancer heterogeneity, this study has some limitations. First, we only collected and analyzed regulators involved in histone acetylation and methylation. Some less prevalent or newly reported histone modification types, such as crotonylation [37], propionylation [38], butyrylation [38], and β-hydroxybutyrylation [39], are also reported to be linked with cancer. Second, we only focused on the transcriptional levels of histone acetylation and methylation regulators and did not integrate other omics data affecting gene expression to classify patients, such as copy number variations, gene mutations, and DNA methylation, meaning that the subtypes analyzed in this study are biased. Third, the various histone modification residues, which were also determinants of the biological functions of histone modifications [4], were not included in this study. Fourth, the method of interpreting the HM_score and the appropriate cutoff values need to be standardized to ensure that the role of this scoring model can be validated in future prospective studies. Last but not least, since the acquisition of ZEB2 comes from CRISPR screening of cell lines in vitro, its role in vivo still needs to be further verified through prospective clinical trials.

## Conclusion

In conclusion, this study comprehensively evaluated the clinical behavior, molecular, and genetic factors associated with histone modifier expression patterns and consequently demonstrated several important insights on how tumor heterogeneity is generated and enhanced by mechanisms underlying epigenetic disorders, as well as proposed promising and effective opportunities for therapeutic intervention. In addition, the HM_score we developed was a clinically valuable tool for identifying patients in the HMC4 cluster precisely by individually quantifying histone modification status and predicting patient survival and chemotherapeutic benefit, thus providing more precise therapeutic guidance in colon cancer in the future.

## Methods

### Public data preparation

The procedure for data analysis was compiled into a flowchart (Additional file 8: Fig. S7). Public transcriptome data on colon cancer samples were retrospectively collected from the GEO (http://www.ncbi.nlm.nih.gov/geo/) and TCGA-COAD (https://cancergenome.nih.gov/) datasets. The demographic and clinical information were retrieved using the “GEOquery” package for GEO datasets or downloaded from the University of California Santa Cruz Xena database (https://xenabrowser.net). The following clinical information was collected: patients’ age, sex, TNM stage, primary tumor site, and chemotherapy performance. The endpoint analyzed in this study was RFS, defined as the interval between the date of diagnosis and date of tumor relapse, and OS, defined as the interval between the date of diagnosis and death. Besides transcriptome data, we also downloaded the somatic mutation data (MAF files, MuTect2 Variant Aggregation and Masking) of patients specimens from ten different tumor types using “TCGAbiolinks” packages to explore the genetic mutation landscape of histone modification regulators. Patient selection criteria for establishing patient cohorts of molecular typing and scoring model development and transcriptome data processing methods are described in Additional file 9: Materials and Methods.

### RNA sequencing of samples from the Sun Yat-sen University Cancer Center cohort

This study was approved by the Nanfang Hospital Ethics Review Board. Thirty fresh samples histologically diagnosed with nonmetastatic colon cancer at the SYSUCC (Guangzhou, China) were included, and RNA extraction and sequencing were performed as described previously [40]. The count values of RNA-sequencing data were transformed using the “voom” algorithm after gene symbol transformation (based on Ensembl ID) in order to convert count data to values similar to those resulting from microarrays [41].

### Identification of histone modifier expression patterns by consensus clustering

The unsupervised clustering (K-means) method was used to identify different histone modifier expression patterns and classify patients for further analysis. A consensus clustering algorithm was used to evaluate clustering stability and select the optimal cluster number using the R package “ConsensusClusterPlus” with the following settings: max*K* = 10, reps = 1000, pItem = 0.95, and pFeature = 1 [42].

### Histone modification score generation

To develop a histone modification score to individually quantify the histone modification status, we first analyzed the differential expressed genes (DEGs) among distinct histone modifier expression patterns in the integrated meta-GEO and TCGA-COAD transcriptional profiling using the “limma” package. The adjusted p value for multiple testing was calculated using the Benjamini–Hochberg correction. The significance criterion for DEGs was set as an absolute “Log2FC” value > 0.5 and an adjusted *p* value < 0.01. Specifically, the DEGs that up- or downregulated in the HMC4 cluster were selected and termed as gene cluster A (801 DEGs upregulated in the HMC4) and cluster B (202 DEGs downregulated in the HMC4), respectively. The Boruta algorithm was employed for the dimension reduction of the gene cluster A and gene cluster B, respectively, using the “Boruta” package (settings: doTrace = 2, maxRuns = 100, ntree = 500) to screen out the most informative genes. The final score was defined as: HM_score = the average expression of final determined gene cluster A—the average expression of final determined gene cluster B.

### Fluorouracil-response prediction

The fluorouracil response of clinical samples was assessed using the R package “pRRophetic,” which implemented a built-in ridge regression model [43] and was qualified as the area under the dose–response curve (AUC), with lower AUC values indicating higher sensitivity to fluorouracil. Further details are provided in Additional file 9: Materials and Methods.

### Biological process and tumor microenvironment characteristics analysis

The biological process and tumor microenvironment characteristics analysis were performed as previously described [19]. Briefly, we utilized GSVA analysis comprising the gene set files of “hallmark gene sets” with the R package “GSVA” to measure biological process activity. An ssGSEA implemented in the “GSVA” R package was used to generate the infiltration scores of the TME cells. The special feature gene panels for marking immune cells [44], stromal cells [45], exhausted T cells [22], and tertiary lymphoid structure [23] were curated from the published literature. The abundance of each cell type was represented by an enrichment score of the gene set in a sample outputted by ssGSEA analysis based on gene expression profiles.

### Cell culture, cell transfection, and MTT assay

Cell culture, cell transfection, and MTT assay were performed as described previously [46]. Further details are provided in Additional file 9: Materials and Methods.

### Genome-wide CRISPR/Cas9 knockout library screen.

The human GeCKO v2 CRISPR library A and library B were used to generate a mutant cell pool for high throughput screening. The criteria for screening candidate sgRNAs were: (1) The average count values of candidate sgRNA in both the fluorouracil-treated group and the vehicle group were greater than 1000; (2) the absolute “Log2FC” value calculated by difference analysis of sgRNA level between the vehicle group and fluorouracil-treated group was more than 0.5. Further details on this matter are provided in Additional file 9: Materials and Methods.

### Connectivity Map analysis

A Connectivity Map analysis was performed to explore the potential molecular targets and specific compounds that could be used for chemosensitization of patients with HMC4 via an online tool (https://clue.io/). A total of 300 DEGs with the most significant fold changes (150 DEGs upregulated and 150 DEGs downregulated in the HMC4 cluster) were entered into the Connectivity Map database following the instructions provided by the website. In this study, the enrichment score generated by Connectivity Map analysis was set to < − 97 and < − 95 for the significant threshold of molecular targets and chemical compounds, respectively.

### Statistical analysis

Statistical analysis was performed using R software version 3.6.0 or SPSS version 25.0 (IBM Corp., Armonk, N.Y., USA). The two-tailed Student’s t test, Mann–Whitney *U* test, Kruskal–Wallis test, one-way ANOVA test, Fisher’s exact test, Pearson’s correlation test, Spearman’s rank correlation test, Cox regression hazard model, and Kaplan–Meier method with the log-rank test were used where necessary. All *p* values were two-tailed, and statistical significance was set to *p* < 0.05 unless noted otherwise. Details are provided in Additional file 9: Materials and Methods.

## Supplementary Information

**Additional file 1:** Supplementary tables.

**Additional file 2: Fig. S1.** Mutations of the “Complex of Proteins Associated with Set1” gene family. **A**–**D** Lollipop diagrams of the landscape of KMT2D (**A**), KMT2C (**B**), KMT2B (**C**), and SETD1B (**D**) mutation positions. (**E**) Boxplot of immune cell infiltration of “Complex of Proteins Associated with Set1” mutation and non mutation groups in the TCGA-COAD cohort. Boxes represent 25–75% of values, lines in boxes represent median values, whiskers represent 1.5 interquartile ranges, and black dots represent outliers. (**F**) Sankey diagram of “Complex of Proteins Associated with Set1” mutations in groups with different molecular subtypes in the TCGA-COAD cohort. MT, mutant type; WT, wild type; CMS, consensus molecular subtypes; MSI, microsatellite instability; MSS, microsatellite stability.

**Additional file 3: Fig. S2.** Alterations of histone modification regulators in the TCGA pan-cancer cohort. **A**–**D** Detailed heatmap of alteration frequencies (left) and mutation rates (right) in members of histone acetyltransferases (**A**), histone deacetylases (**B**), histone methyltransferases (**C**), and histone demethylases regulators (**D**) across solid tumors in the TCGA pan-cancer cohort.

**Additional file 4: Fig. S3.** The optimal cluster number as determined by the consensus clustering algorithm. **A**–**C** Consensus matrixes (left) of patients with colon cancer for *k* = 4 and line graphs (right) of relative changes in the area under the CDF curve according to the cluster number in the meta-GEO (**A**), TCGA-COAD (**B**), and integrated GEO and TCGA-COAD cohorts (**C**). **D** Heatmaps of the enrichment of histone modification regulators in different histone modifier expression patterns in the meta-GEO (left), TCGA-COAD cohort (middle), and integrated GEO and TCGA-COAD cohorts (right). (E–F) Boxplot of distribution of histone modifier expressions among different histone modifier expression patterns in the meta-GEO (**E**) and TCGA-COAD cohorts (**F**). Boxes represent 25–75% of values, lines in boxes represent median values, whiskers represent 1.5 interquartile ranges, and black dots represent outliers. *p < 0.05, **p < 0.01, ***p < 0.001; ns, not significant.

**Additional file 5: Fig. S4.** Distribution of SIIS value among HMC clusters. Boxplot of SIIS value in the four studied histone modifier expression patterns in the meta-GEO (left) and TCGA-COAD (right) cohorts. Boxes represent 25–75% of values, lines in boxes represent median values, whiskers represent 1.5 interquartile ranges, and black dots represent outliers. *p < 0.05, **p < 0.01, ***p < 0.001.

**Additional file 6: Fig. S5.** Association between HM_score and histone modifier expression patterns. **A**–**C** (left) Boxplot of the HM_score values of the different modification clusters in the meta-GEO (**A**), TCGA-COAD (**B**), and SYSUCC (**C**) cohorts. Boxes represent 25–75% of values, lines in boxes represent median values, whiskers represent 1.5 interquartile ranges, and black dots represent outliers. **A**–**C** (right) receiver operating characteristics curve of the HM_score model for distinguishing patients in the HMC4 cluster from those who are not in the meta-GEO (**A**), TCGA (**B**), and SYSUCC cohorts (**C**). Ref, reference; AUC, area under curve.

**Additional file 7: Fig. S6.** ZEB2 overexpression experiment. Dose–response curves of SW480 (left) and HCT116 cells (right) transfected with empty vectors or ZEB2 plasmid after fluorouracil treatment for 24 h. The mean ± standard deviation of the three replicates of each time point is shown. *p < 0.05, **p < 0.01, ***p < 0.001.

**Additional file 8: Fig. S7.** The flowchart of data analysis procedure.

**Additional file 9:** Supplementary Materials and Methods.

## Data Availability

The public data used in this study are available at: GSE17536 (http://www.ncbi.nlm.nih.gov/geo/query/acc.cgi?acc=GSE17536); GSE33113 (http://www.ncbi.nlm.nih.gov/geo/query/acc.cgi?acc=GSE33113); GSE37892 (http://www.ncbi.nlm.nih.gov/geo/query/acc.cgi?acc=GSE37892); GSE38832 (http://www.ncbi.nlm.nih.gov/geo/query/acc.cgi?acc=GSE38832); GSE39582 (http://www.ncbi.nlm.nih.gov/geo/query/acc.cgi?acc=GSE39582); and TCGA-COAD (https://xenabrowser.net/datapages/?cohort=TCGA Colon Cancer (COAD). The SUSYCC colon cancer dataset generated and analyzed during the current study is not publicly available but is available from the corresponding author on reasonable request.

## Abbreviations

AJDC: Adjuvant chemotherapy
CI: Confidence interval
CIMP: CpG island methylator phenotype
CIN: Chromosome instability
CMS: Consensus molecular subtypes
DEGs: Differentially expressed genes
EMT: Epithelial–mesenchymal transition
FC: Fold change
GSVA: Gene set variation analysis
ITH: Intratumoral heterogeneity
MT: Mutant type
NEO: Neoantigen
NR: Nonresponse
OS: Overall survival
R: Response
Ref: Reference
RFS: Relapse-free survival
ROC: Receiver operating characteristic
ssGSEA: Single-sample gene set enrichment analysis
SYSUCC: Sun Yat-sen University Cancer Center
TEX: T cell exhaustion
TLS: Tertiary lymphoid structure
TMB: Tumor mutation burden
TME: Tumor microenvironment
WT: Wild type
