# Supplementary figures and images for: Cross talk between acetylation and methylation regulators reveals histone modifier expression patterns posing prognostic and therapeutic implications on patients with colon cancer
Source: Clin Epigenetics. 2022 May 23;14:70. doi: 10.1186/s13148-022-01290-y (PMC9128235; doi:10.1186/s13148-022-01290-y)

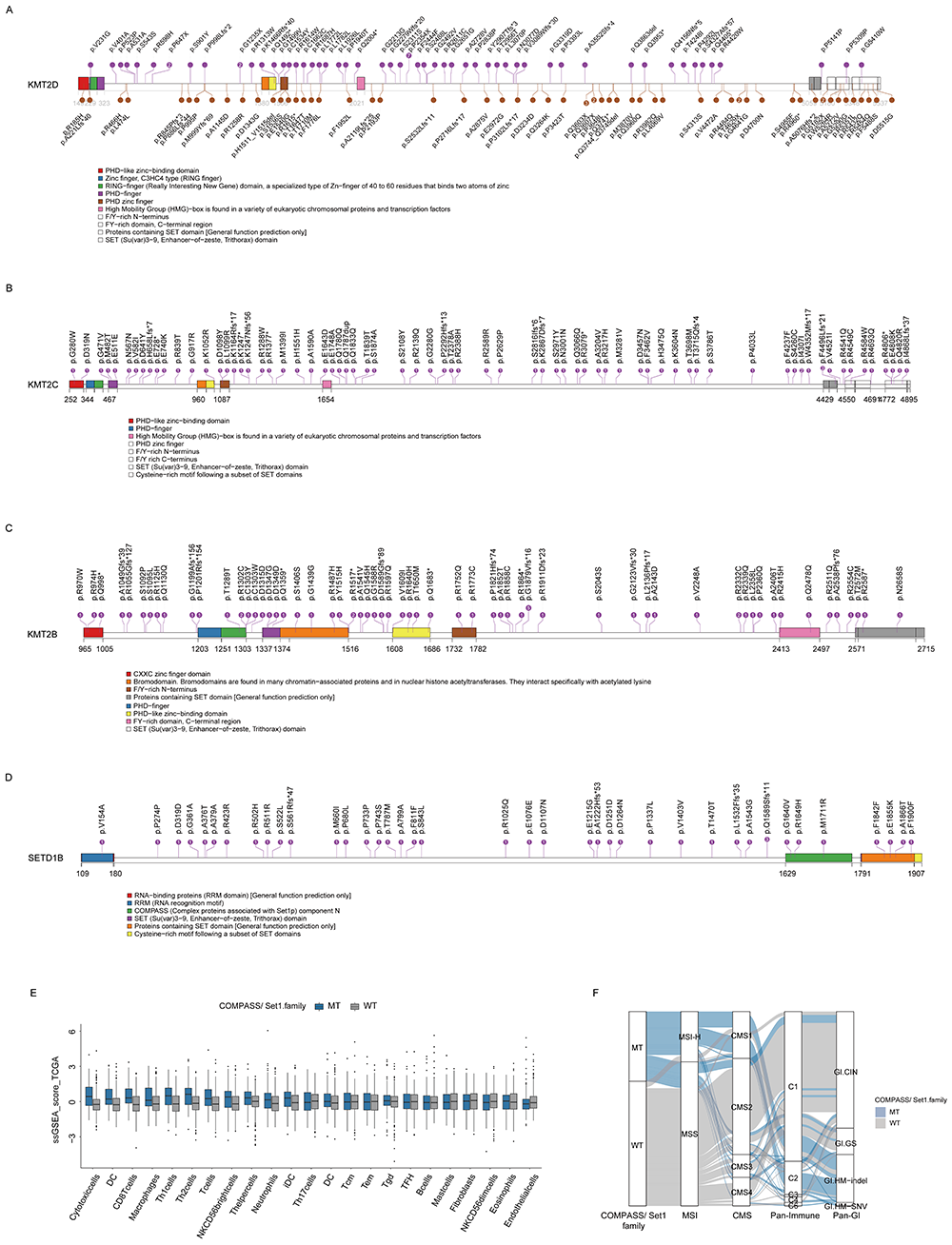

Supplement: Supplementary file 2 — Additional file 2: Fig. S1. Mutations of the “Complex of Proteins Associated with Set1” gene family. A–D Lollipop diagrams of the landscape of KMT2D (A), KMT2C (B), KMT2B (C), and SETD1B (D) mutation positions. (E) Boxplot of immune cell infiltration of “Complex of Proteins Associated with Set1” mutation and non mutation groups in the TCGA-COAD cohort. Boxes represent 25–75% of values, lines in boxes represent median values, whiskers represent 1.5 interquartile ranges, and black dots represent outliers. (F) Sankey diagram of “Complex of Proteins Associated with Set1” mutations in groups with different molecular subtypes in the TCGA-COAD cohort. MT, mutant type; WT, wild type; CMS, consensus molecular subtypes; MSI, microsatellite instability; MSS, microsatellite stability. [file 13148_2022_1290_MOESM2_ESM.tif]

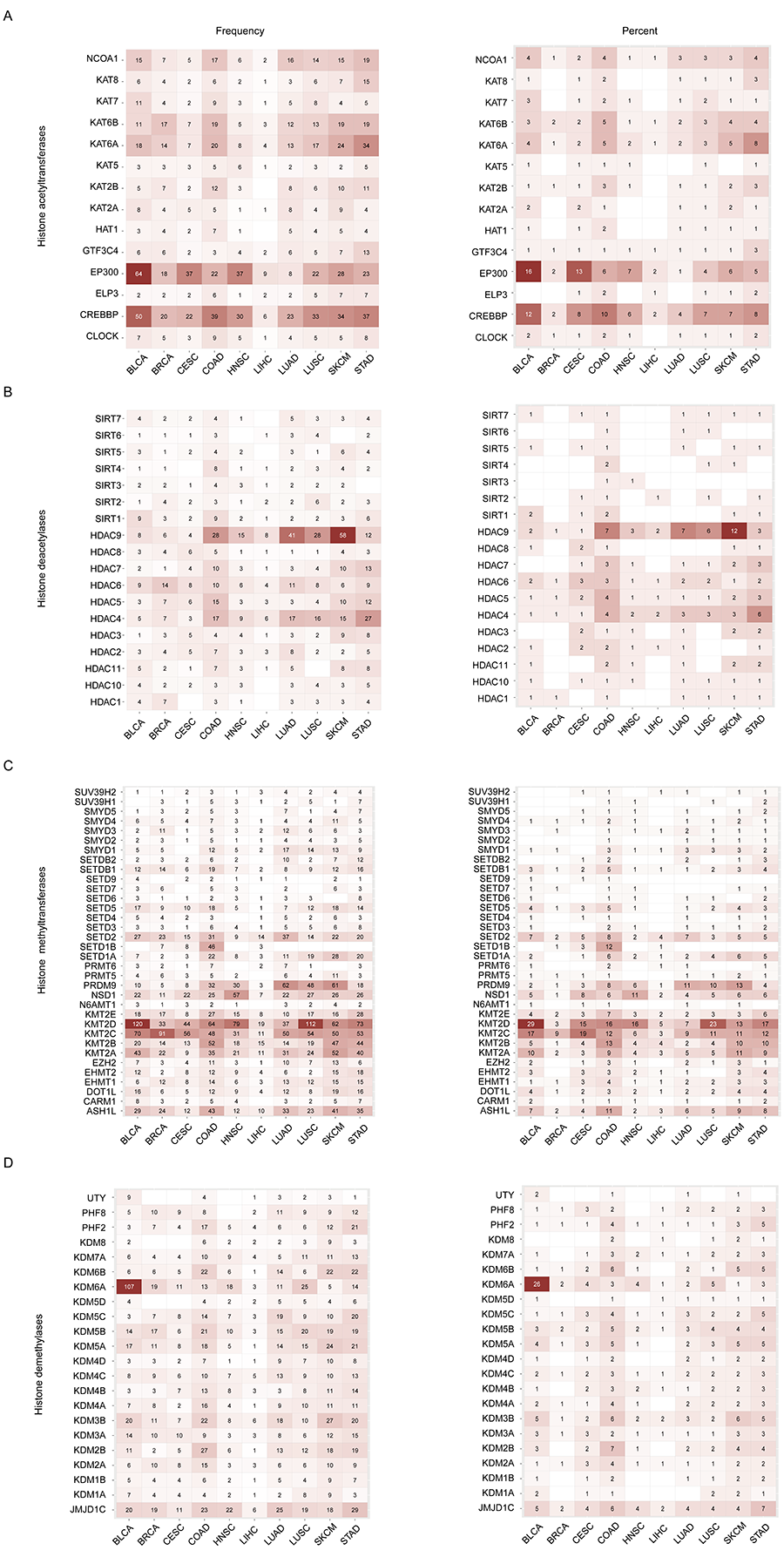

Supplement: Supplementary file 3 — Additional file 3: Fig. S2. Alterations of histone modification regulators in the TCGA pan-cancer cohort. A–D Detailed heatmap of alteration frequencies (left) and mutation rates (right) in members of histone acetyltransferases (A), histone deacetylases (B), histone methyltransferases (C), and histone demethylases regulators (D) across solid tumors in the TCGA pan-cancer cohort. [file 13148_2022_1290_MOESM3_ESM.tif]

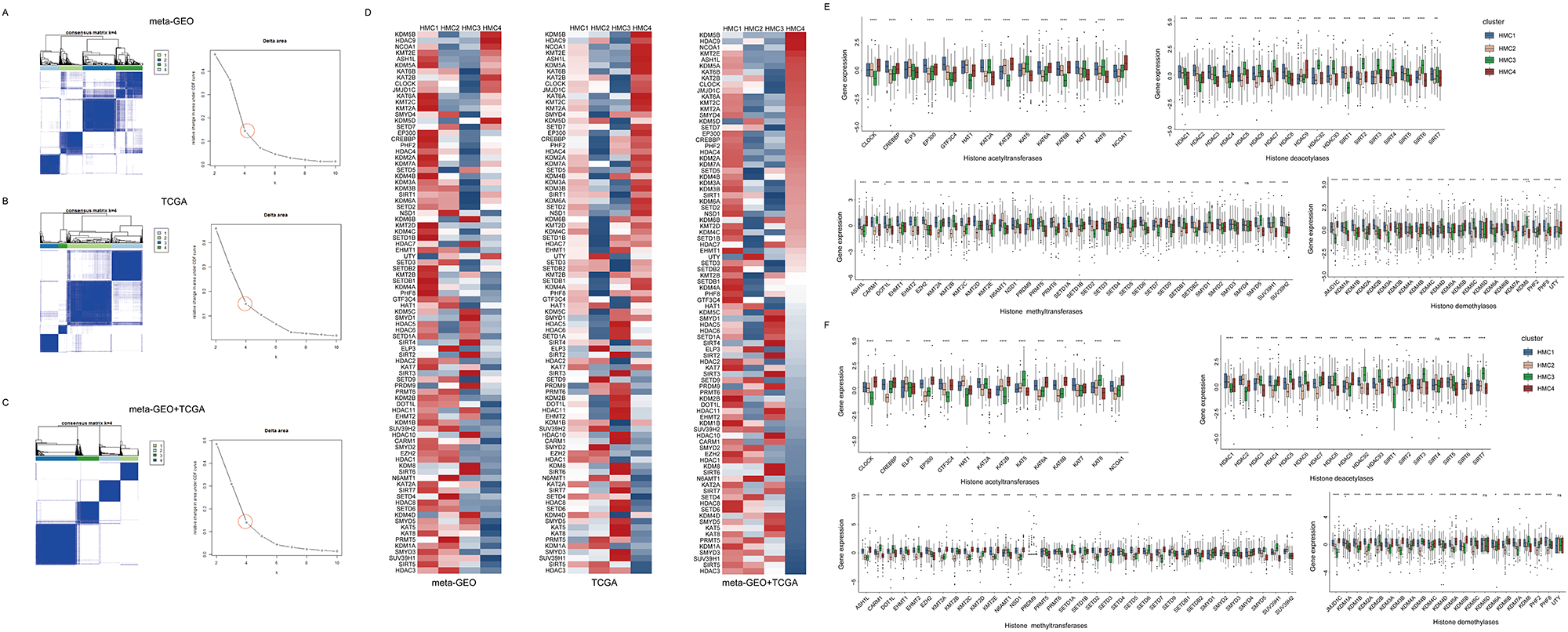

Supplement: Supplementary file 4 — Additional file 4: Fig. S3. The optimal cluster number as determined by the consensus clustering algorithm. A–C Consensus matrixes (left) of patients with colon cancer for k = 4 and line graphs (right) of relative changes in the area under the CDF curve according to the cluster number in the meta-GEO (A), TCGA-COAD (B), and integrated GEO and TCGA-COAD cohorts (C). D Heatmaps of the enrichment of histone modification regulators in different histone modifier expression patterns in the meta-GEO (left), TCGA-COAD cohort (middle), and integrated GEO and TCGA-COAD cohorts (right). (E–F) Boxplot of distribution of histone modifier expressions among different histone modifier expression patterns in the meta-GEO (E) and TCGA-COAD cohorts (F). Boxes represent 25–75% of values, lines in boxes represent median values, whiskers represent 1.5 interquartile ranges, and black dots represent outliers. *p < 0.05, **p < 0.01, ***p < 0.001; ns, not significant. [file 13148_2022_1290_MOESM4_ESM.tif]

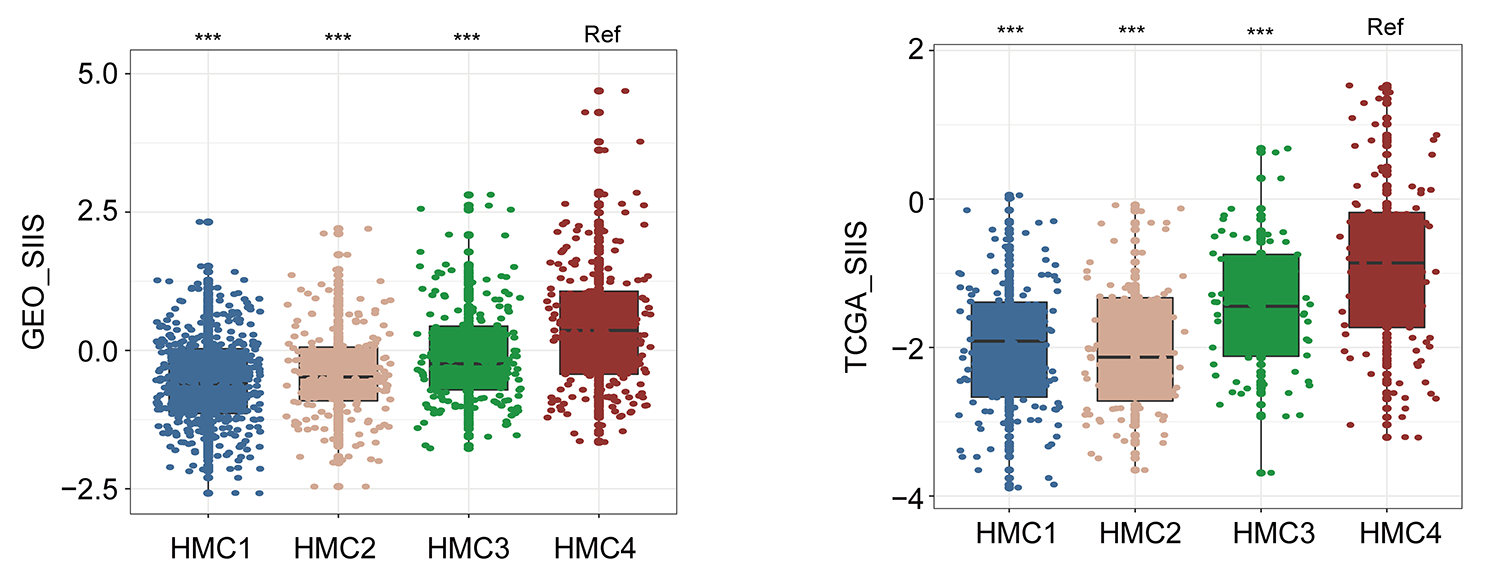

Supplement: Supplementary file 5 — Additional file 5: Fig. S4. Distribution of SIIS value among HMC clusters. Boxplot of SIIS value in the four studied histone modifier expression patterns in the meta-GEO (left) and TCGA-COAD (right) cohorts. Boxes represent 25–75% of values, lines in boxes represent median values, whiskers represent 1.5 interquartile ranges, and black dots represent outliers. *p < 0.05, **p < 0.01, ***p < 0.001. [file 13148_2022_1290_MOESM5_ESM.tif]

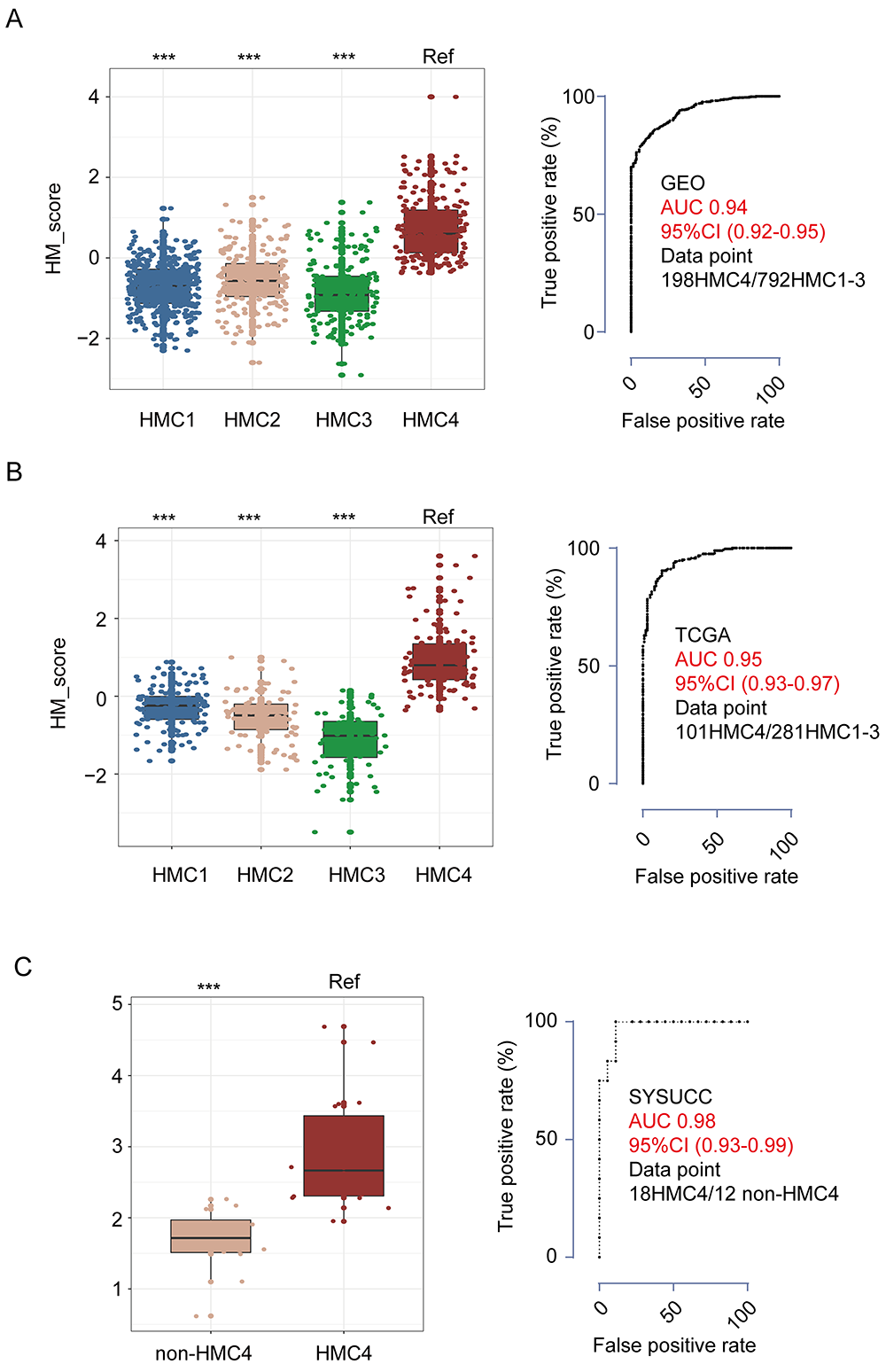

Supplement: Supplementary file 6 — Additional file 6: Fig. S5. Association between HM_score and histone modifier expression patterns. A–C (left) Boxplot of the HM_score values of the different modification clusters in the meta-GEO (A), TCGA-COAD (B), and SYSUCC (C) cohorts. Boxes represent 25–75% of values, lines in boxes represent median values, whiskers represent 1.5 interquartile ranges, and black dots represent outliers. A–C (right) receiver operating characteristics curve of the HM_score model for distinguishing patients in the HMC4 cluster from those who are not in the meta-GEO (A), TCGA (B), and SYSUCC cohorts (C). Ref, reference; AUC, area under curve. [file 13148_2022_1290_MOESM6_ESM.tif]

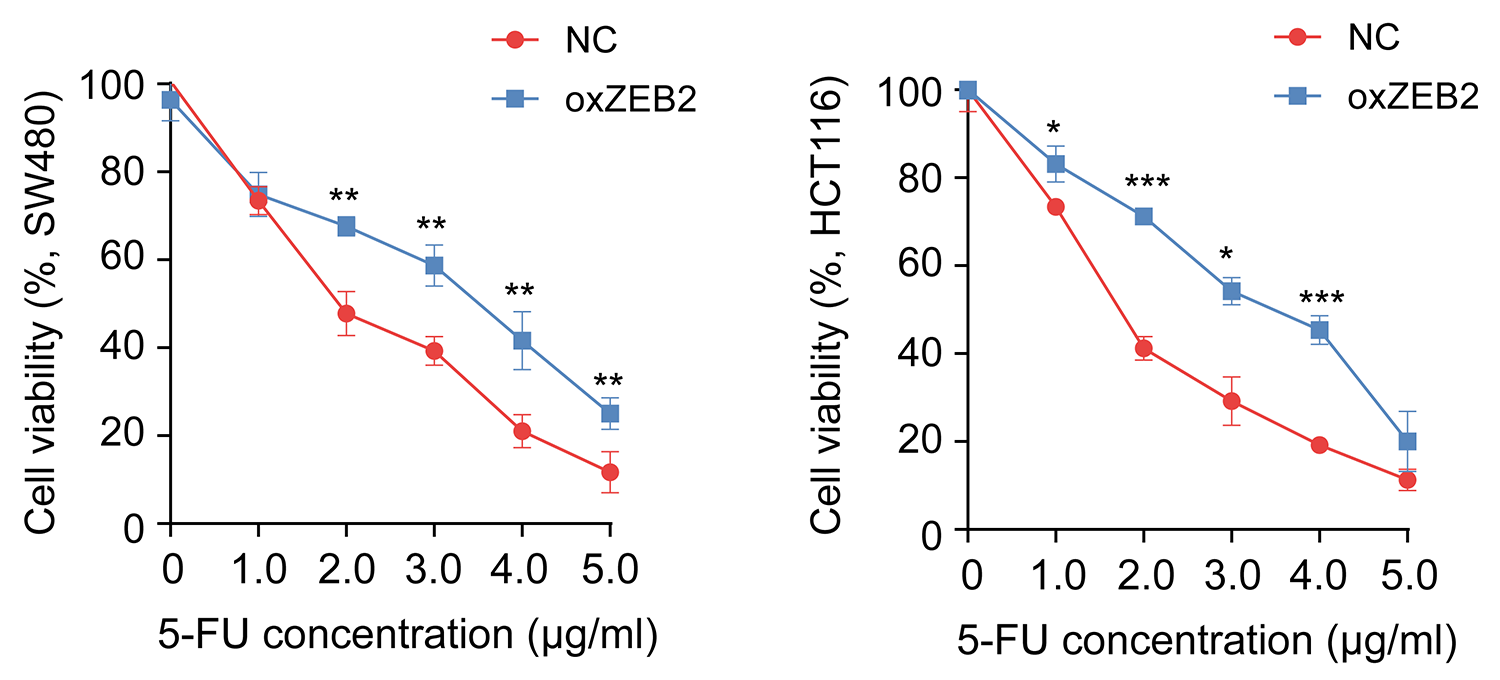

Supplement: Supplementary file 7 — Additional file 7: Fig. S6. ZEB2 overexpression experiment. Dose–response curves of SW480 (left) and HCT116 cells (right) transfected with empty vectors or ZEB2 plasmid after fluorouracil treatment for 24 h. The mean ± standard deviation of the three replicates of each time point is shown. *p < 0.05, **p < 0.01, ***p < 0.001. [file 13148_2022_1290_MOESM7_ESM.tif]

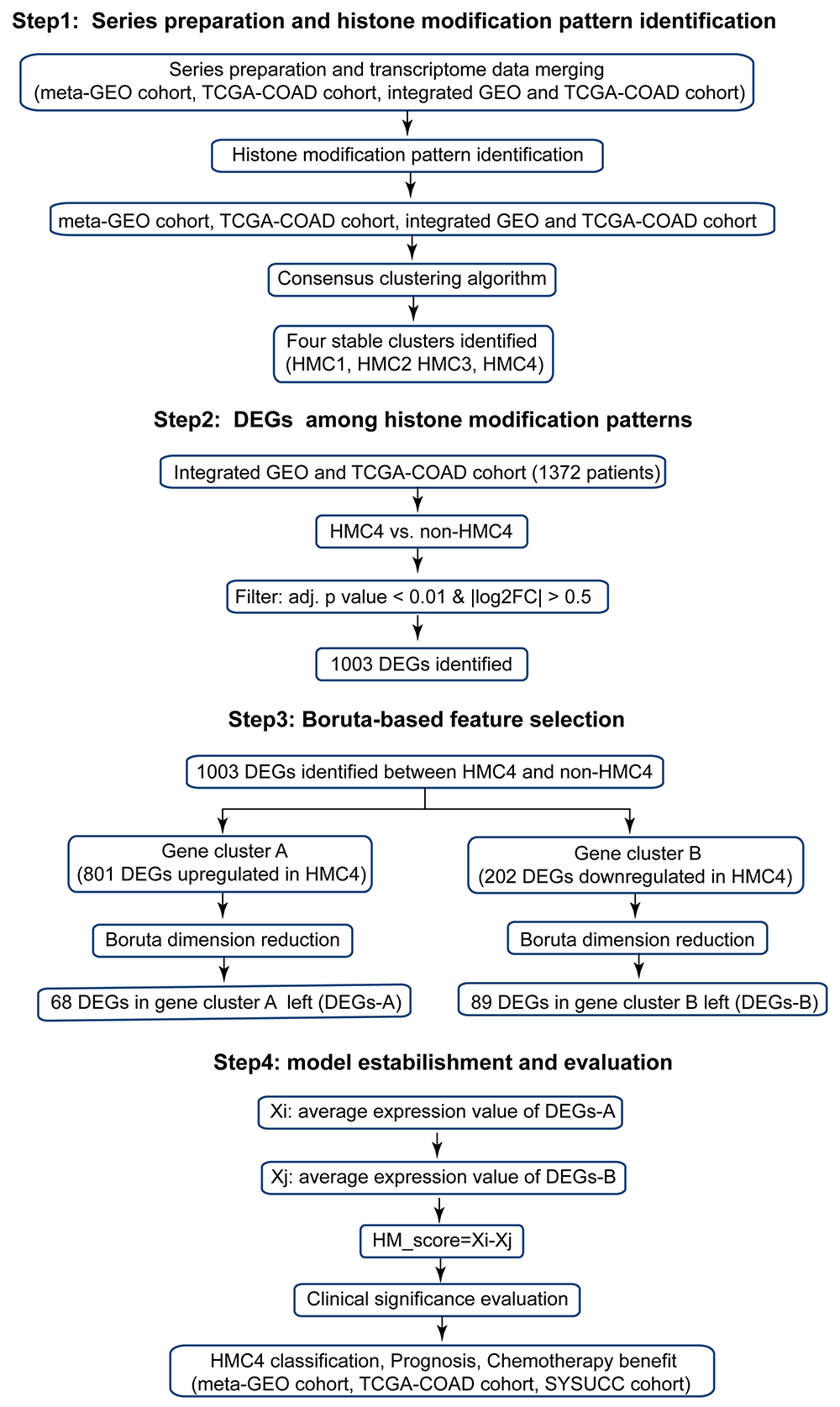

Supplement: Supplementary file 8 — Additional file 8: Fig. S7. The flowchart of data analysis procedure. [file 13148_2022_1290_MOESM8_ESM.tif]
