# Supplementary material for: Cross talk between acetylation and methylation regulators reveals histone modifier expression patterns posing prognostic and therapeutic implications on patients with colon cancer
Source: Clin Epigenetics. 2022 May 23;14:70. doi: 10.1186/s13148-022-01290-y (PMC9128235; doi:10.1186/s13148-022-01290-y)
Supplement: Supplementary file 9 — Additional file 9: Supplementary Materials and Methods. [file 13148_2022_1290_MOESM9_ESM.docx]

**Supplementary Materials and Methods**

**1. Patients’ selection criteria for establishing patient cohorts of molecular typing and scoring model development**

The GEO was searched for eligible colon cancer datasets for molecular typing and scoring model development that fulfilled the following criteria: samples were hybridized to the Affymetrix HG-U133 Plus 2.0 (GEO accession number GPL570) platforms; more than 50 stage I–III colon cancer patients were included in each dataset; and information on the TNM stage was available. For samples from the TCGA-COAD database, we also ruled out samples of patients with metastatic colon cancer (stage IV). Finally, a total of 1372 patients were enrolled for identifying histone modifier expression pattern and histone score modeling analysis, including those from the GSE17538 (N = 176), GSE33113 (N = 90), GSE37892 (N = 130), GSE38832 (N = 92), GSE39582 (N = 502), and TCGA-COAD (The Cancer Genome Atlas-Colon Adenocarcinoma, N = 382) datasets (Additional file 1-Tables S4-5).

**2. Transcriptome data processing of GEO and TCGA-COAD database**

For microarray data retrieved from the GEO database, since all samples involved in the downloaded colon cancer datasets were hybridised to Affymetrix HG-U133 Plus 2.0, the raw “CEL” files of microarray data were retrieved and all renormalized using a robust multiarray averaging method with “affy” and “simpleaffy” packages as we previously described([1](#_ENREF_1)). For samples of the TCGA-COAD database, we downloaded level three “HTSeq-Counts” data of eligible samples from the University of California Santa Cruz Xena database (https://xenabrowser.net), and the RNA sequencing data was transformed using the “voom” algorithm after gene symbol transformation in order to convert count data to values similar to those resulting from microarrays([2](#_ENREF_2)). The “ComBat” algorithm of the “SVA” Package was applied to reduce the likelihood of batch effects from non-biological technical biases when merging the transcriptome data from different database.

**3. Fluorouracil response prediction**

The drug sensitivity information of cancer cell lines were obtained from the Cancer Therapeutics Response Portal (https://portals.broadinstitute.org/ctrp), which contains sensitivity data for 481 compounds in 835 cell lines, and cell sensitivity to drugs is qualified as the area under the dose-response curve (AUC), with lower AUC values indicating higher sensitivity to treatment. Cell lines derived from hematopoietic and lymphoid tissues were removed. Drugs with more than 80% of non-missing data were retained, and then K-nearest neighbor using a Euclidean metric was applied for missing data imputation. Finally, the AUC of fluorouracil of each clinical sample from the GSE39582 and TCGA-COAD datasets was calculated with R package ‘pRRophetic’, which has a built-in ridge regression model that was used to predict the chemotherapy response of clinical samples based on their transcriptomic profiles.

Samples with AUC value in the upper third of the whole cohort (GSE39582 or TCGA-COAD) or greater than the median (SYSUCC) would be classified into the fluorouracil-nonresponse group, while samples with AUC value in the lower third (GSE39582 or TCGA-COAD) or less than the median (SYSUCC) would be classified into the fluorouracil response group.

**4. CRISPR/Cas9 knockout library screen**

The mutant cell pool was treated with vehicle or fluorouracil to enable positive and negative screening([3](#_ENREF_3), [4](#_ENREF_4)). In the presence of fluorouracil, cells carrying sgRNA targeting fluorouracil resistance genes will be negatively selected in the mutant cell pool, and their corresponding sgRNA will also be depleted in the library that can be determined by high-throughput sequencing. In detail, the SW480 cells transduced with human GeCKO (Genome-Scale CRISPR Knock-Out) lentiviral A and lentiviral B were selected with blasticidin (MDBio, D0120601) for 15 days to generate a mutant cell pool. Then, cells were treated with 2 μg/mL fluorouracil or vehicle for 72 h and the treatment media were removed. The residual cells in fluorouracil-treated group were allowed to regrow to confluency, which is called a round of screening. After three rounds of screening, the fluorouracil-resistant cells and cells in the vehicle group were collected for genomic DNA extraction and deep sequencing analysis. The count value of sgRNAs was transformed using the “voom” algorithm, and differentially enriched sgRNAs between fluorouracil- and vehicle-treated groups were analyzed using the “limma” package. The adjusted p-value for multiple testing was calculated using the Benjamini–Hochberg correction.

**5. Cell culture and cell transfection**

Cancer cell lines, including HCT116 and SW480, were routinely maintained in Roswell Park Memorial Institute (RPMI) 1640 medium with 10% fetal bovine serum and cultured at 37 °C under 5% CO_2_. All cell lines were cultured at 37 °C under 5% CO_2_. For cell transfection, tumor cells were seeded in 6-well plates until 30%–40% density was reached. Thereafter, 50 μM siRNA (GEMA, Suzhou, China), 5 μL Lipofectamine 2000 transfection reagent, and Opti-MEM were transfected into the cells according to the manufacturer’s instructions.

**6. Methylthiazolyl-tetrazolium (MTT) assay**

Cells were seeded in 96-wells plates at a density of 6,000/wells for 48 hr treatments. After the treatment, MTT solution with a final concentration of 0.5 mg/mL was added to each well and incubated at 37°C for 4 hr. Then, the crystals were dissolved with 150 μL of dimethyl sulfoxide each well. Finally, the optical density was detected at 492 nm using Microplate spectrophotometer (SpectraMax M5).

**7. Statistical analysis**

For comparisons of two groups, statistical significance was estimated via Student’s t-tests for normally distributed variables, or via Mann-Whitney *U* test for non-normally distributed variables. Kruskal-Wallis (non-parametric) and one-way ANOVA tests (parametric methods) were used for comparisons of more than two groups. When comparing the difference of categorical data, Fisher’s exact tests were used. Pearson’s correlation and Spearman’s rank correlation coefficient was used to evaluate the correlation between two parameters when appropriate. Survival analyses including log-rank tests, Kaplan–Meier method, uni- and multivariate Cox analyses were performed using the “survival” package. The best cut-off values for each continuous prognostic marker were calculated using the “survminer” package.

**References**

1. Zhou R, Zeng D, Zhang J, Sun H, Wu J, Li N, et al. A robust panel based on tumour microenvironment genes for prognostic prediction and tailoring therapies in stage I-III colon cancer. EBioMedicine. 2019;42:420-30.

2. Ali HR, Chlon L, Pharoah PD, Markowetz F, Caldas C. Patterns of Immune Infiltration in Breast Cancer and Their Clinical Implications: A Gene-Expression-Based Retrospective Study. PLoS medicine. 2016;13(12):e1002194.

3. Sanjana NE, Shalem O, Zhang F. Improved vectors and genome-wide libraries for CRISPR screening. Nature methods. 2014;11(8):783-4.

4. Shalem O, Sanjana NE, Hartenian E, Shi X, Scott DA, Mikkelson T, et al. Genome-scale CRISPR-Cas9 knockout screening in human cells. Science. 2014;343(6166):84-7.
